# Hypothetical mechanisms driving physical activity levels in ethnic minority groups living in Europe: a systematically identified evidence-based conceptual systems model

**DOI:** 10.1186/s12966-024-01626-2

**Published:** 2024-08-07

**Authors:** Alexia D. M. Sawyer, Frank van Lenthe, Carlijn Kamphuis, Enrique Garcia Bengoechea, Aleksandra Luszczynska, Laura Terragni, Kevin Volf, Gun Roos, Catherine Woods, Sarah Forberger, Marie Scheidmeir, Lars Jørun Langøien, Agnieszka Neumann-Podczaska, Katarzyna Wieczorowska-Tobis, Karien Stronks

**Affiliations:** 1 Medical Research Council Epidemiology Unit, University of Cambridge, Cambridge, UK; 2 Department of Public Health, Erasmus Medical Center, Erasmus University Rotterdam, Rotterdam, The Netherlands; 3 Department of Interdisciplinary Social Science, Utrecht University, Utrecht, The Netherlands; 4 Department of Physical Education and Sport Sciences, Physical Activity for Health Research Centre, Health Research Institute, University of Limerick, Limerick, Ireland; 5 Center for Applied Research On Health Behavior and Health, SWPS University, Wroclaw, Poland; 6 Department of Nursing and Health Promotion, Oslo Metropolitan University, Oslo, Norway; 7 Centre for Welfare and Labour Research, Oslo Metropolitan University, Oslo, Norway; 8 Department of Prevention and Evaluation, The Leibniz Institute for Prevention Research and Epidemiology – BIPS, Bremen, Germany; 9 Department of Health Science, University of York, York, UK; 10 Department of Health Psychology, Johannes Gutenberg University Mainz, Mainz, Germany; 11 Department of Reviews and Health Technology Assessments, Norwegian Institute of Public Health, Oslo, Norway; 12 Department of Palliative Care, Poznan University of Medical Sciences, Poznan, Poland; 13 Department of Public and Occupational Health, Amsterdam UMC, University of Amsterdam, Amsterdam, The Netherlands

## Abstract

**Background:**

In Europe, physical activity levels tend to be lower in ethnic minority groups than the general population. Interventions and policies based on research examining isolated determinants of physical activity have had limited success in increasing physical activity levels. This study used systems dynamics theory and the capability approach theoretical framework to develop a conceptual model of how individual characteristics, institutional and physical environments and the migration context may interact to promote or hinder physical activity in ethnic minority groups living in Europe.

**Methods:**

A systematic update of Langøien et al.’s 2017 review of the determinants of physical activity in ethnic minority groups living in Europe was conducted. Our target population included individuals of all ages who reported a familial migration background from any low- and middle-income countries or belonging to minority indigenous population in Europe. Outcomes pertaining to non-work related physical activity of light, moderate or vigorous intensity performed in any setting were included. Included studies provided an evidence base from which to derive the causal loop diagrams comprising our conceptual model. Sub-system causal loop diagrams were interpreted in co-author review sessions to explicate non-linear system mechanisms, such as reinforcing and balancing feedback loops.

**Results:**

Forty-one studies were identified, of which the majority was qualitative. The conceptual model consisted of 4 causal loop diagrams relating to psychosocial constructs; sociocultural constructs; health and health communication and social and material resources, in interaction with environmental/migration context. Four hypothetical mechanisms were identified, e.g. hypothesizing that participation in organised activities leads to increased self-efficacy, thereby enabling further participation.

**Conclusions:**

This study contributes an evidence-based conceptual systems model which elucidates how low levels of physical activity in ethnic minority groups in Europe could be supported by reinforcing and balancing mechanisms involving factors relating to physical and institutional environments, migration context and individuals. A pluralistic approach to literature review, integrating complexity methods such as CLDs into more conventional systematic literature review, supports novel insights into how factors could interact to support persistently low levels of activity, moving beyond the identification of potential relationships between isolated factors to indicating the ways in which these relationships are sustained and could be modified by intervention or policy.

**Supplementary Information:**

The online version contains supplementary material available at 10.1186/s12966-024-01626-2.

## Background

Performing 150–300 min of moderate to vigorous physical activity a week has myriad physical and mental health benefits, including reducing the risk of non-communicable illness such as cardiovascular disease, cancer, stroke and depression and risk of and recovery from infectious illness [1–4]. However, on average across European countries, 48% of adults report performing no non-work-related physical activity, and only 32% report performing at least 150 min of non-work related activity per week [5]. Moreover, the distribution of physical activity is not equal across the population in Europe. As elsewhere globally, people with an ethnic minority identity who, at a group level, have been collectively minoritised by social processes [6], are more likely to report lower levels of physical activity [7–9].

Interventions informed by previous research exploring the determinants of physical activity in ethnic minority groups have shown mixed effects and, despite implementation of these interventions, low levels of physical activity are still reported in ethnic minority groups [10–12]. In part, this could be because it is the interaction *among* determinants which is driving emergent patterns of unequally-distributed physical activity. This includes the interactions between environmental factors and individual-level factors, e.g. in the case of urban neighbourhood parks being used differently by men and women, leading to differential associations between park use and physical activity [13].

**Fig. 1:**
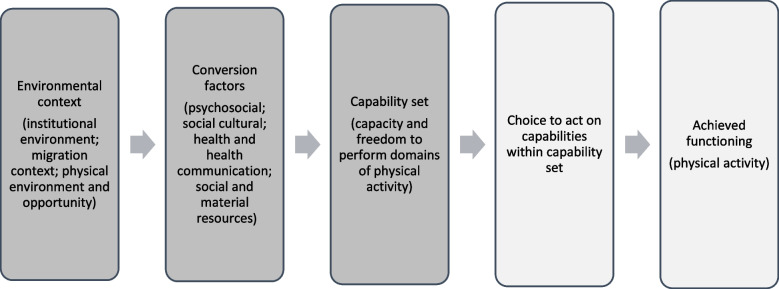
Application of the capability approach in the current study. Note: Boxes in shaded darker grey are the focus of the current study

The capability approach by Amartya Sen and Martha Nussbaum provides a theoretical framework to conceptualise how shared physical or institutional environments may be differentially experienced by groups owing to psychological, social and cultural factors and access to material resources [14–16]. According to the capability approach, ‘achieved functionings’ such as being physically active, are only realised when relevant ‘capabilities’ (i.e. the capacity and freedom to be or do something) distinguish certain functionings (i.e. doing or being something) as more or less possible, meaningful or desirable to individuals [17]. Figure 1 illustrates how the capability approach can be applied to physical activity in ethnic minority groups, whereby environmental and individual or social conversion factors (i.e. the factors determining the degree to which a resource can be turned into a capability) interact to produce a capability set that makes physical activity more or less likely for individuals in this group.

Accordingly, the focus on determinants of behaviour is shifted upstream to the *determinants of capabilities* required to achieve that behaviour, or ‘functioning’ [17]. The value of taking a capabilities approach to study physical activity promotion lies in this pivoting away from improving outcomes directly towards improving *the conditions needed to achieve those outcomes*. A systematic review of natural experiments examining the impact of physical environmental changes on physical activity in the general population found variable levels of effect, with 17 of 26 studies reporting a positive effect on physical activity levels [18]. However, effectiveness was shown to differ across factors such as the personal characteristics of study participants [18].

System dynamics theory is congruous with the capability approach framework as it acknowledges that emergent outcomes, such as physical activity levels, are the result of the interaction between factors situated at different levels of space (e.g. environmental, social, individual) and time (e.g. historical influences, life-course influences, present day influences) in a complex adaptive system [19]. Within a systems approach, methods such as causal loop diagramming can be used to present an integrated view of multiple factors with non-linear relationships between one another, unpacking how certain outcomes could emerge from – and be sustained by – reinforcing and stablishing mechanisms constructed from these relationships.

As physical activity is a ‘best buy’ in public health [20], we need to understand how to intervene to create physical, social and institutional (i.e. educational, professional, religious or social organisations) environments in Europe which equitably support diverse populations to be physically active. To effectively create such environments, it is necessary to comprehensively and holistically understand the causal pathways (or mechanisms)—including interactions between environmental and individual factors—through which unequal levels of physical activity emerge.

This study aimed to understand the mechanisms through which institutional and physical environments and the migration context enable and disable physical activity in ethnic minority groups living in Europe. To achieve this, we used systematically-identified empirical evidence to develop causal loop diagrams (CLDs) which can visually delineate the emergence of a set of physical activity capabilities belonging to individuals in ethnic minority groups in Europe. This set can be considered an average for a heterogeneous population in terms of country of origin (e.g. African or Asian countries), socioeconomic status (e.g. high and low income), and acculturation (e.g. assimilation versus marginalisation).

It was our objective to develop a conceptual systems-based model of the determinants of physical activity for ethnic minority groups in Europe, not to develop a deterministic model of the determinants of physical activity for individuals as we deemed this neither possible nor desirable, acknowledging that there will be a high degree of heterogeneity between individuals [21, 22]. Nonetheless, we anticipate that by unpacking the emergence of an *average* set of capabilities we can generate new insights into the potential causal pathways through which contextual factors can promote or hinder opportunities for physical activity in groups who tend to perform insufficient activity, and thereby inform future intervention and policy development.

## Methods

### Study design

Following the methodology developed in Sawyer et al. [23], a systematic review was conducted in order to obtain an evidence base from which to derive the CLDs. The protocol for the review was registered with PROSPERO prior to conducting the review (CRD42021244927). Using the selected evidence base, a conceptual systems map was constructed as a series of sub-system CLDs to explicate non-linear system mechanisms, such as reinforcing and balancing feedback loops. Unlike a traditional systematic review, the aim of this review was not to include, appraise and report all evidence published on this topic. Rather, it was to draw on a comprehensive, non-biased set of peer-reviewed publications to develop a conceptual model of the complex influences on physical activity in a specific population sub-group of people from ethnic minority groups.

### Systematic review of the literature

The systematic review of the literature was an update of Langøien, Terragni, Rugseth et al.’s 2017 review of the determinants of physical activity in ethnic minority groups living in Europe [24]. The same search protocol was used for the updated search. A pluralist approach to evidence selection (i.e. inclusion of different study designs) suited the current review’s exploratory research question and is useful in building an evidence base from which to understand complex relationships between multiple factors [23, 25]. The approach used in the current review draws on: conventional methods for systematically reviewing peer-reviewed literature; methods for reviewing the literature from a realist perspective to understand mechanisms underlying relationships [26]; and emerging methods and approaches used to conduct systems-based literature reviews [23, 27].

The boundaries of the system under study (i.e. the scope of the research question) as well as the choice for Langøien et al.’s systematic review informed the inclusion and exclusion criteria and the population, outcomes and determinants of interest [24]. Given the central aim of synthesising the current evidence of underlying mechanisms of physical activity in ethnic minority groups living in Europe, we used the following inclusion and exclusion criteria:

### Population

Our targeted population included children and adolescents (2–18 years), adults and older adults (18 + years) living in Europe, with a familial migration background from any low- and middle-income countries (including the former Eastern European Bloc countries) or from minority indigenous populations in Europe.

### Outcomes

All outcomes pertaining to non-work related physical activity of light, moderate or vigorous intensity performed in any setting were included. Physical activity is defined as energy expenditure resulting from bodily movement created by skeletal muscles [2].

### Determinants

Results from Langøien et al.’s [24] systematic review were used in a concept mapping exercise by Holdsworth et al. [28], resulting in 8 clusters of factors of physical activity: psychosocial, institutional environment, political environment, social and cultural environment, physical environment and opportunity, social and material resources, health and health communication, migration context. Determinants related to these clusters were considered relevant to the research question.

### Inclusion criteria

• Studies with a target population of (an) ethnic minority group(s): “immigrants and their offspring/populations of immigrant background (not differentiating on their migration status) from low and middle income countries, population groups from the former Eastern European Bloc countries who migrate to other parts of Europe and minority indigenous populations in Europe” [24].
• Studies with a target population of a ‘majority’ ethnic group and (an) ethnic minority group(s) were included where sub-analyses were reported for (an) ethnic minority group(s).
• Children, adolescents and young people, adults, and older adults (age ranges as characterised by study authors);
• Qualitative or quantitative studies using non-experimental study designs or quasi-, controlled or natural experimental study designs; the combination of different study designs enables us to build CLDs and take a systems perspective;
• European setting;
• Studies exploring determinants (independent variables) in relation to migration background and/or ethnicity; i.e. studies must study determinants or mechanisms of physical activity by migration background and/or ethnicity.
• No restrictions on language or date of publication.

### Exclusion criteria

• Studies that only describe (differences in) physical activity levels by migration background and/or ethnicity;
• Studies that examine physical activity in the relationship between disease and migration background and/or ethnicity, without examining determinants of physical activity;
• Studies that examine interaction between determinants of physical activity but not interaction between contextual factors and conversion factors (as described in Fig. 1);
• Literature reviews, position or conceptual papers were excluded from data extraction but included in search in order to conduct reference search;
• Grey literature.

### Search strategy

Six databases (MEDLINE, EMBASE [Ovid], Web of Science, Cochrane Library, CINAHL, PsycINFO [Ovid]) were searched in March 2021 using predefined free-text words and MESH terms, modified for each database. Search terms were taken from Langøien et al. [24]; the only change was the removal of terms relating to sedentary behaviour and the addition of the term ‘physical inactivity’. The search terms for each database are reported in Supplementary File 1. AS performed the search and stored retrieved records using Rayyan QCRI software. Duplicated records were removed prior to screening.

Title, abstract and full-text screening was conducted by AS, MS, SF, KV, ANP and KWT. At each stage of screening, records were screened independently by two researchers and any disagreements between reviewers were assessed by a third researcher; further disagreements were resolved through discussion.

### Data extraction

Included studies were stored in EndNote. Data were extracted by AS, CBMK, SF and MS. Data extraction was independently completed for 10% of included studies to check consistency and accuracy in the extraction process. Study characteristics were extracted for the studies which were identified through the updated search (i.e. were not included in Langøien et al. [24]) and checked for the studies which were included in the original review by Langøien et al. [24].

For each study, factors that were examined in direct relation or indirect relation (e.g. effect modification) to our outcomes of interest were extracted using their original labelling. Factors were sorted using the categories from Holdsworth et al. [28]. A new category was added by the reviewer if no suitable category was available; an example of a new category is ‘use of motorised private transport’. Where appropriate, new categories added by different reviewers were conflated by AS, retaining the original factor labels to enable double-review.

Following the categorisation of all factors, a selection of factors originally drawn from Holdsworth et al. [28] were relabelled to better reflect the included factors (e.g. ‘opportunities in life’ was relabelled ‘skilled work opportunities’ as factors within this category all related to employment opportunities). KS conducted a double-review of: variable names included in each category; creation and conflation of new categories; and the definition or relabelling of categories. Any disagreements were resolved in discussion with AS.

To document the reported relationships between factors, all relevant examined associations between factors were reported in a spreadsheet which had all factor categories listed in the first column and first row to create a matrix. In the appropriate cell (e.g. cell X by Y to report the relationship between a factor in category X and a factor in category Y), the reviewer noted: the study reference, the original factor name (rather than the category label), the original framing of the factor (e.g. ‘low levels of social support’ or ‘high levels of social support’), the direction of the studied relationship (the effect of X on Y, Y on X, a correlation, or a modifying effect), the reported significance of the relationship according to the measure of significance used in the original study (e.g. p-value, qualitative interpretation; reported as: significant, non-significant or a trend) and the physical activity outcome assessed in relation to these factors. Relationships between a single factor and a physical activity outcome was not recorded as the objective of this study was to examine the relationships *between* determinants of physical activity. A hypothetical entry could read: “ [19] low levels of parental support for physical activity ➔ (significant) low levels of motivation for physical activity ➔ (significant) reduced interest in physical activity”. Each reviewer received detailed guidance on data extraction and populated their own spreadsheet. Spreadsheets were collated by AS at the end of data extraction.

### Quality assessment

As the aim of this literature review was to identify and elucidate mechanisms rather than make conclusive judgements (e.g. on clinical effectiveness), we took a pluralistic approach, synthesising qualitative and quantitative evidence from multiple types of study design. The quality assessment checklist by Kmet, Lee & Cook [29] allowed assessments of risk of bias in primary research using a range of study designs. Where physical activity in ethnic minority groups was a partial focus of a selected study, quality assessments were only conducted on the parts of the study which pertained to our research question. To ensure consistency, quality assessments were performed for all included studies, not just those obtained in the updated search. Quality assessments were independently conducted by AL, CW, EGB, AS and GR.

### Data synthesis and CLD development

All factors and associations which, on balance across the selected evidence, were qualitatively or statistically significant were entered into a CLD using Kumu software. Factors (or CLD ‘nodes’) were colour-coded according to their overarching category [28]: ‘psychosocial’, ‘sociocultural’, ‘health and health communication’, ‘migration context’, ‘social and material resources’, ‘institutional environment’ and ‘physical environment and opportunity’. The direction and polarity of associations were recorded using connection arrows.

In line with the capability approach, factors within the categories of ‘institutional environment’, ‘migration context’ and ‘physical environment and opportunity’ were conceptualised as contextual factors. Factors within all other categories were conceived as characteristics of the target group and therefore conceptualised as conversion factors. It was theorised that the interaction between contextual factors and conversion factors produce an emergent set of capabilities which do or do not enable physical activity (Fig. 1). As previously outlined, this interaction was the primary interest for the current study.

Sub-system CLDs were created to understand the hypothetical relationship between the environmental context and conversion factors. Feedback loops and mechanisms within these CLDs were analysed in order to understand how this relationship could lead to the emergence of particular capabilities for physical activity. The choice to act on capabilities to achieve a certain functioning (i.e. physical activity outcome) was not in the scope of this study and therefore not within the boundaries of the CLD (light grey boxes in Fig. 1).

Separate sub-system CLDs were created for each category of conversion factors. Exogenous contextual factors, defined as factors that did not have incoming connections, were excluded. Because they were not connected to other factors, they cannot contribute to potential system dynamics of interest. By screening the results from the automatic detection of feedback loop using Kumu software, feedback loops involving a conversion factor and at least two but no more than 5 other (conversion or contextual) factors were identified for interpretation by co-authors with expertise in the relevant field. Feedback loops comprising more than 6 nodes were deemed too complex for qualitative interpretation.

### CLD interpretation

An initial review session of all 4 sub-system CLDs was attended by AS, KS and FvL and used to check for possible human errors in the translation of the evidence to the CLD. This check involved inspecting factor labels and the direction and polarity of connection arrows. Subsequently, a separate review session for each sub-system CLD was held in June 2023, involving co-authors with demonstrable expertise in the field (e.g. expertise in psychosocial influences on physical activity). Sessions were held online and typically lasted 90 min. Prior to the review session, reviewers were asked to scrutinise a document including the sub-system CLD and each identified feedback loop presented separately. Reviewers were asked to inspect the CLD and feedback loops for errors and consider their initial interpretation of feedback loops.

During the review sessions, reviewers were first asked to report any identified errors or raise any queries about unexpected present or missing connection arrows. AS used the collated data extraction spreadsheet which noted linkage between connection arrows and the primary studies to check errors and queries in real time; where it was not possible to resolve the issue during the session, AS noted queries for further examination. Next, reviewers examined each feedback loop in turn, discussing interpretations and supporting evidence. Initially, AS presented each feedback loop as a set of connection arrows, drawing on the supporting evidence from the primary studies to explain the association between each node (to facilitate this, studies were cited for each connection arrow using the Notes feature in Kumu). The review groups then interpreted the feedback loops as presenting reinforcing or balancing mechanisms, where a reinforcing feedback loop indicates increasing growth or decline in the level of included nodes over time (e.g. increasing levels of social support) and a balancing feedback loop indicates stabilisation in the level of included nodes over time (e.g. stable levels of social support). Missing evidence was also discussed at reviewer sessions, identifying one systematic review; the primary studies included in this review were latterly screened and excluded by AS. The following co-authors took part in the review sessions: environmental, migration and psychosocial factors sub-system: AS, KV, EGB, ANP, CW; environmental, migration and sociocultural factors sub-system: AS, AL, GR, SF, EGB; environmental, migration and health and health communication factors sub-system: AS, LL, LT, KWT, EGB; environmental, migration and social and material resources sub-system: AS, EGB, CBMK, SF.

## Results

**Fig. 2:**
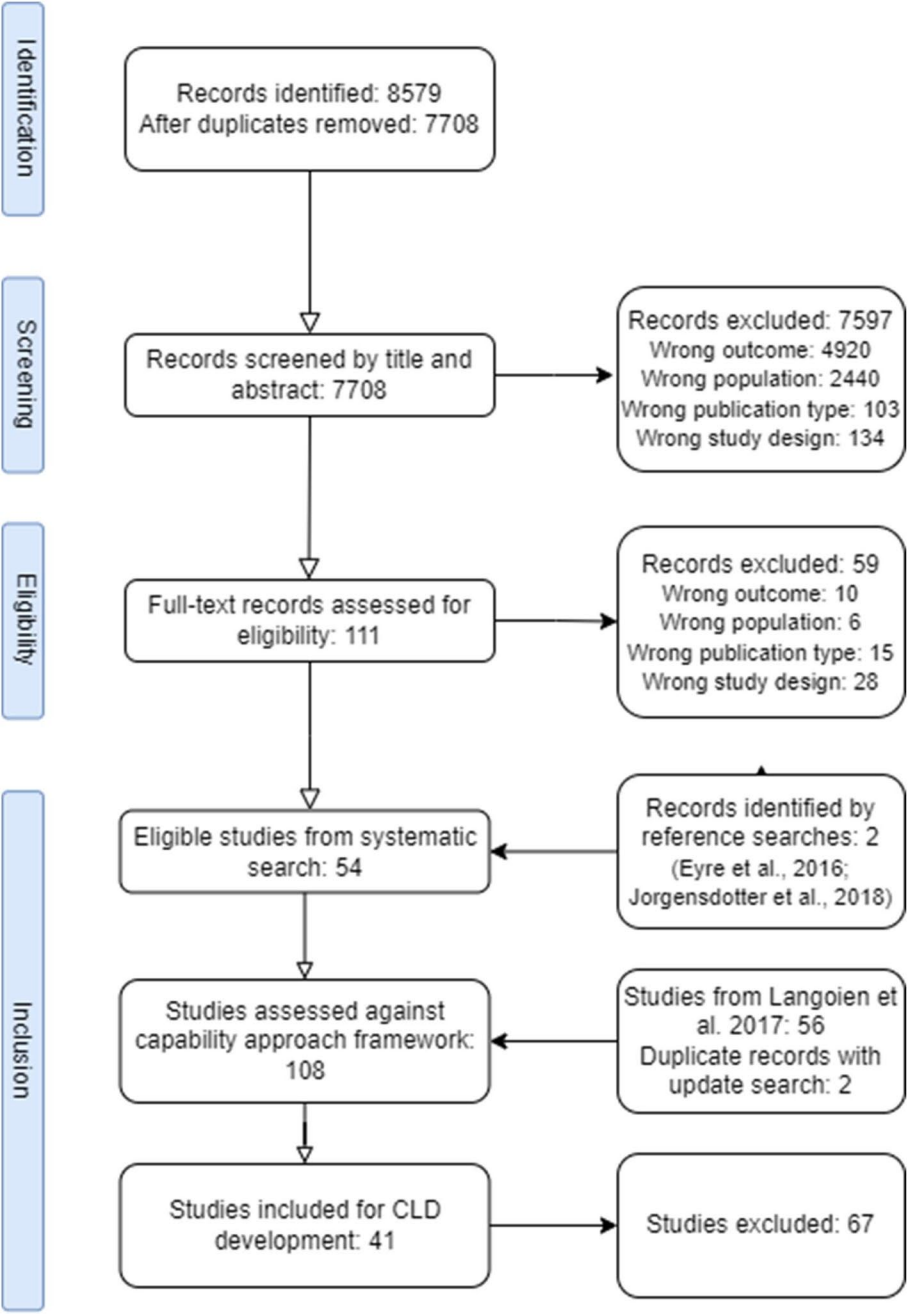
Flowchart of selection process for included studies

The initial search identified 7708 individual studies. Of these, 54 studies were identified for consideration against the capability approach framework, adding to 54 studies from the review by Langøien et al. [24]. Of these 108 studies, 41 studies examined the interaction between contextual factors and conversion factors (Fig. 1) and were thus used for development of the CLDs. Figure 2 shows the flow of studies.

The characteristics of included studies are presented in Supplementary File 2. The target population of most studies was adult (adults: 22 studies; children or young people: 10 studies; older adults: 5 studies; age group of target population not specified: 4 studies) and female or mixed sex (females only: 20 studies; mixed sex: 20 studies; males only: 1 study). Most studies primarily characterised their sample by ethnicity, race or nationality (35 studies) rather than religion (Muslim with a migration background: 6 studies). Nearly three-quarters of the studies used qualitative methods (qualitative: 29 studies; quantitative: 8 studies, mixed methods: 4 studies), and only 2 studies reported data from experimental studies.

### Sub-system feedback loops and overarching hypothetical mechanisms

Twenty-six feedback loops were identified across the four sub-system CLDs, with 17 of the feedback loops coming from the environment, migration and sociocultural sub-system. Most feedback loops were reinforcing loops (19 loops) rather than balancing loops (7 loops) and many of the feedback loops interacted with one another (i.e. included factors that were present in more than one feedback loop), supporting the interpretation of overarching mechanisms for each sub-system. Furthermore, all feedback loops included at least one contextual factor and at least one conversion factor: 24 loops included a physical environment and opportunity contextual factor; 12 loops included a migration contextual factor and 9 loops included an institutional environment contextual factor (numbers add up to more than 26 as many loops included more than one category of contextual factor). This illustrates the importance of the interaction between contextual factors and conversion factors in driving the mechanisms which may determine the capability to be physically active.

**Fig. 3:**
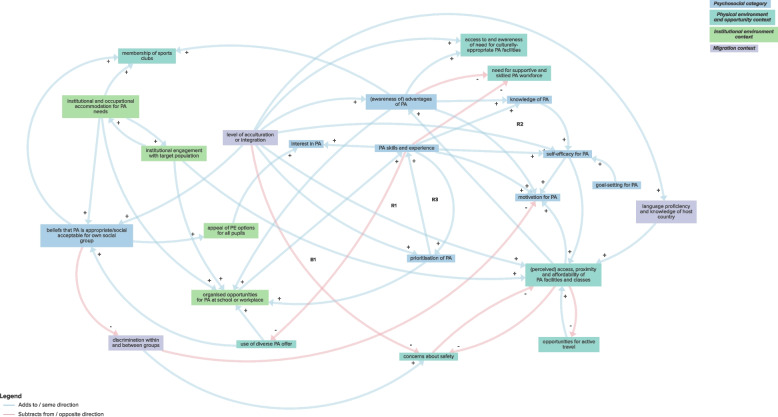
Sub-system CLD exploring interaction between environmental, migration and psychosocial factors

The sub-system CLD exploring the interaction between environmental, migration and psychosocial factors included 1 balancing and 3 reinforcing feedback loops comprising 3–5 factors which were interpreted as contributing to an overarching potential mechanism whereby physical activity was promoted through opportunities to ‘learn by doing’ (Fig. 3; Table 1; Supplementary File 3). Feedback loops revealed that exposure to organised opportunities for physical activity promoted skills, experience and awareness of the benefits of physical activity, leading to increased self-efficacy and prioritisation of physical activity which enabled further participation in physical activity. For example, reinforcing feedback loop R2 describes how increased awareness of the benefits of physical activity can lead to greater knowledge of activity concepts and health-promoting guidelines. This enhanced knowledge can boost self-efficacy for physical activity and make facilities and classes appear more welcoming and geographically accessible (e.g. by active transportation). As a result, higher attendance at physical activity facilities and classes can further raise awareness of the advantages of physical activity, both tacitly and explicitly. A lack or lower level of physical activity opportunities can therefore lead to lower levels of skills, experiences and awareness of the benefits of physical activity and so on.

**Table 1:** Sub-system feedback loops and overarching mechanisms

Sub-system	Feedback loops	Hypothetical mechanisms
Environmental, migration and psychosocial factors	B1: PA skills and experience ➔- use of diverse PA offer ➔ + organised opportunities for PA at school or workplace ➔ + PA skills and experience	*Overarching mechanism:* *Physical activity is promoted through opportunities to “learn by doing”:*
	R1: PA skills and experience ➔ + prioritisation of PA ➔ + organised opportunities for PA at school or workplace ➔ + PA skills and experience	Underlying mechanisms: 1. Strengthening PA skills and experience through increased PA participation 2. Prioritisation of PA leading to increased PA skills and experience 3. Bolstering awareness of PA advantages through increased access to PA facilities and classes
	R2: awareness of advantages of PA ➔ + knowledge of PA ➔ + self-efficacy for PA ➔ + perceived access, proximity and affordability of PA facilities and classes ➔ + awareness of advantages of PA	
	R3: PA skills and experience ➔ + self-efficacy for PA ➔ + perceived access, proximity and affordability of PA facilities and classes ➔ + awareness of advantages of PA ➔ + organised opportunities for PA at school or workplace ➔ + PA skills and experience	
Environmental, migration and sociocultural factors	R1: impact of gender roles, expectations and obligations ➔ + concerns about safety ➔- perceived access, proximity and affordability of PA facilities and classes ➔- impact of gender roles, expectations and obligations	*Overarching mechanism:* *Sociocultural influences present barriers to participation in physical activity; increasing access can overcome these barriers over time:*
	R2a: impact of gender roles, expectations and obligations ➔- level of acculturation and integration ➔ + perceived access, proximity and affordability of PA facilities and classes ➔- impact of gender roles, expectations and obligations R2b: impact of gender roles, expectations and obligations ➔- level of acculturation and integration ➔- concerns about safety ➔- perceived access, proximity and affordability of PA facilities and classes ➔- impact of gender roles, expectations and obligations R2c: impact of gender roles, expectations and obligations ➔- level of acculturation and integration ➔ + language proficiency and knowledge of host country ➔ + perceived access, proximity and affordability of PA facilities and classes ➔- impact of gender roles, expectations and obligations R2d: impact of gender roles, expectations and obligations ➔- level of acculturation and integration ➔ + opportunities for social connection ➔ + perceived access, proximity and affordability of PA facilities and classes ➔- impact of gender roles, expectations and obligations R2e: impact of gender roles, expectations and obligations ➔- level of acculturation and integration ➔ + access to and awareness of need for culturally-appropriate PA facilities ➔ + opportunities for social connection ➔ + perceived access, proximity and affordability of PA facilities and classes ➔- impact of gender roles, expectations and obligations R3a: level of acculturation and integration ➔ + access to and awareness of need for culturally-appropriate PA facilities ➔ + opportunities for social connection ➔ + level of acculturation and integration R3b: level of acculturation and integration ➔ + (perceived) access, proximity and affordability of PA facilities and classes ➔ + opportunities for social connection ➔ + level of acculturation and integration R3c: level of acculturation and integration ➔- concerns about safety ➔- perceived access, proximity and affordability of PA facilities and classes ➔ + opportunities for social connection ➔ + level of acculturation and integration R3d: level of acculturation and integration ➔ + language proficiency and knowledge of host country ➔ + perceived access, proximity and affordability of PA facilities and classes ➔ + opportunities for social connection ➔ + level of acculturation and integration R3e: level of acculturation and integration ➔- impact of gender roles, expectations and obligations ➔ + concerns about safety ➔- perceived access, proximity and affordability of PA facilities and classes ➔ + opportunities for social connection ➔ + level of acculturation and integration	Underlying mechanisms: 1. Concerns about safety, low levels of acculturation, low language proficiency and knowledge of host country, time and energy constraints and reduced opportunities for social connection contributing to growing impact of gender roles, expectations and obligations through increasingly reduced access to appropriate PA facilities and classes. 2. High levels of acculturation and integration are bolstered by opportunities for social connection obtained through increased access to (culturally-appropriate) physical activity facilities, supported by fewer concerns about safety and increased language proficiency and knowledge of host country. 3. Time and energy constraints (arising from family and work commitments alongside additional challenges of living in a new country or facing discrimination) reduce participation in workplace physical activity classes and social connection which could otherwise increase access to local physical activity facilities and classes. 4. More organised workplace physical activity classes can lead to reduced time and energy constraints by increasing opportunities for social connection, levels of integration, perceived access of physical activity classes and reducing the time commitments needed to participate in physical activity.
	B1: time and energy constraints ➔- organised opportunities for PA at school and workplace ➔ + opportunities for social connection ➔ + perceived access, proximity and affordability of PA facilities and classes ➔- time and energy constraints	
	R4a: institutional engagement with target population ➔ + (perceived) access, proximity and affordability of PA facilities and classes ➔- impact of gender roles, expectations and obligations ➔ + importance of religious and cultural practices ➔- (perceived) number of school facilities ➔ + institutional engagement with target population	
	R4b: (perceived) number of school facilities ➔ + access to sex-segregated PA facilities and instruction ➔ + (perceived) access, proximity and affordability of PA facilities and classes ➔- impact of gender roles, expectations and obligations ➔ + importance of religious and cultural practices ➔- (perceived) number of school facilities	
	R5a: impact of gender roles, expectations and obligations ➔ + time and energy constraints ➔- organised opportunities for PA at school or workplace ➔ + opportunities for social connection ➔ + perceived access, proximity and affordability of PA facilities and classes ➔- impact of gender roles, expectations and obligations	
	R5b: impact of gender roles, expectations and obligations ➔ + time and energy constraints ➔- organised opportunities for PA at school or workplace ➔ + opportunities for social connection ➔ + level of acculturation and integration ➔- impact of gender roles, expectations and obligations	
	R5c: organised opportunities for PA at school or workplace ➔ + opportunities for social connection ➔ + level of acculturation and integration ➔ + perceived access, proximity and affordability of PA facilities and classes ➔- time and energy constraints ➔- organised opportunities for PA at school and workplace	
Environmental, migration and health and health communication factors	B1a: ill health and pain ➔ + advice and instrumental support from healthcare ➔ + access to sex-segregated PA facilities and instruction ➔ + (perceived) access, proximity and affordability of PA facilities and classes ➔- concerns about safety ➔- physical fitness ➔- ill health and pain	*Overarching mechanism:* *Effective health communication can enable management of health conditions through physical activity:*
	B1b: ill health and pain ➔- mental health ➔ + advice and instrumental support from healthcare ➔ + access to sex-segregated PA facilities and instruction ➔ + (perceived) access, proximity and affordability of PA facilities and classes ➔- concerns about safety ➔- physical fitness ➔- ill health and pain	Underlying mechanism: 1. Poorer physical and co-morbid mental health can be managed through guided use of appropriate physical activity facilities and instruction.
Environmental, migration and social and material resources factors	B1a: (perceived) access, proximity and affordability of PA facilities and classes ➔- concerns about safety ➔ + use of motorised private transport ➔ + (perceived) access, proximity and affordability of PA facilities and classes	*Overarching mechanism:* *Multi-faceted access characteristics influencing the use of motorised transport over active travel:*
	B1b: (perceived) access, proximity and affordability of PA facilities and classes ➔- concerns about safety ➔ + use of motorised private transport ➔- opportunities for active travel ➔ + (perceived) access, proximity and affordability of PA facilities and classes	Underlying mechanism: 1. The use of motorised transport is sustained due to physical activity facilities being perceived as distant or inaccessible through increased concerns about safety and reduced opportunities for active travel to such facilities.
	R1a: (perceived) access, proximity and affordability of PA facilities and classes ➔ + opportunities for active travel ➔- use of motorised private transport ➔ + (perceived) access, proximity and affordability of PA facilities and classes	

*PA* physical activity, *B* balancing feedback loop, *R* reinforcing feedback loop

➔- indicates a negative relationship where increase in the leading variable leads to decrease in the following variable, or decrease in the leading variable leads to increase in the following variable

➔ + indicates a positive relationship where increase in the leading variable leads to increase in the following variable, or decrease in the leading variable leads to decrease in the following variable

**Fig. 4:**
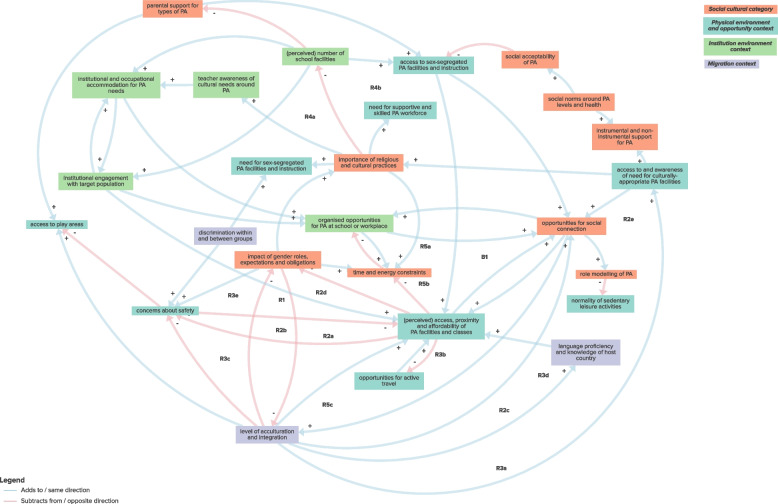
Sub-system CLD exploring interaction between environmental, migration and sociocultural factors

The sub-system CLD exploring the interaction between environmental, migration and sociocultural factors included 1 balancing and 16 reinforcing feedback loops, pointing to potential mechanisms which enable physical activity through factors which can increase opportunities for social connection, or discourage physical activity through factors which can lead to an increased impact of time and energy constraints and gender roles, expectations and obligations (Fig. 4; Table 1; Supplementary File 3). These mechanisms were interpreted as underpinning an overarching hypothetical mechanism whereby sociocultural influences dominant in certain population sub-groups can present barriers to participation in physical activity in a European context, but increasing access to physical activity can overcome these barriers over time. For example reinforcing feedback loop R2d describes how gender roles can lead to lower levels of acculturation and integration in other communities, reducing opportunities for social connection with members of these groups. This limited social connection can result in reduced access to PA facilities and classes, as these resources may be unfamiliar and are not promoted by community members. Consequently, this restricted access can frame such facilities or classes as unsuitable for girls and women, further reinforcing the impact of gender roles on physical activity. However, as specified in reinforcing feedback loop R3a, high levels of integration can mitigate the impact of gender roles, thereby reducing, e.g., safety concerns for women and girls. With fewer safety concerns, access to PA facilities and classes increases, as they are perceived as safe. This enhanced access fosters opportunities for social connections within and between group members, further promoting an individual’s integration within their own and other communities.

**Fig. 5:**
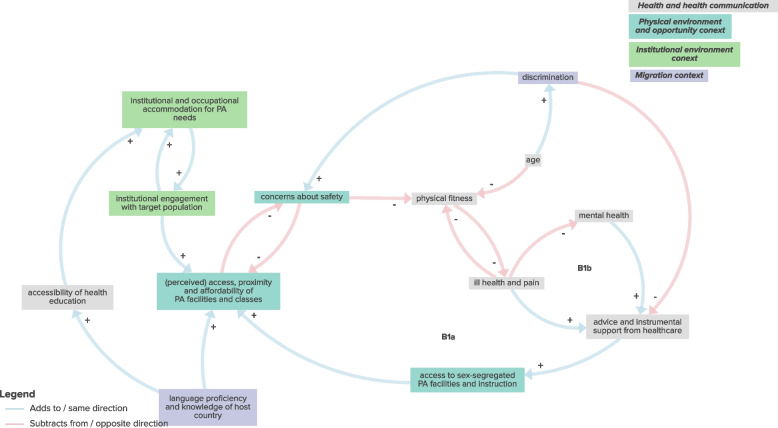
Sub-system CLD exploring interaction between environmental, migration and health and health communication factors

The sub-system CLD exploring the interaction between environmental, migration and health and health communication factors included 2 balancing feedback loops which were interpreted as supporting an overarching hypothetical mechanism whereby effective health communication enables the management of physical and mental health conditions through physical activity (Fig. 5; Table 1; Supplementary File 3). For example, balancing loop B1b illustrates that the increased levels of ill health and pain—as generally seen in ethnic minority groups—increase the likelihood of seeking and/receiving and instrumental support for physical activity from health care providers. This advice and support can enhance access to sex-segregated PA facilities and instruction, which in turn can improve perceived or actual access to general PA facilities and classes in the local area. This increased familiarity and knowledge of the area can alleviate safety concerns, encouraging greater us of local facilities. As a result, individuals may experience improved physical fitness, which helps to manage or prevent ill health and pain.

**Fig. 6:**
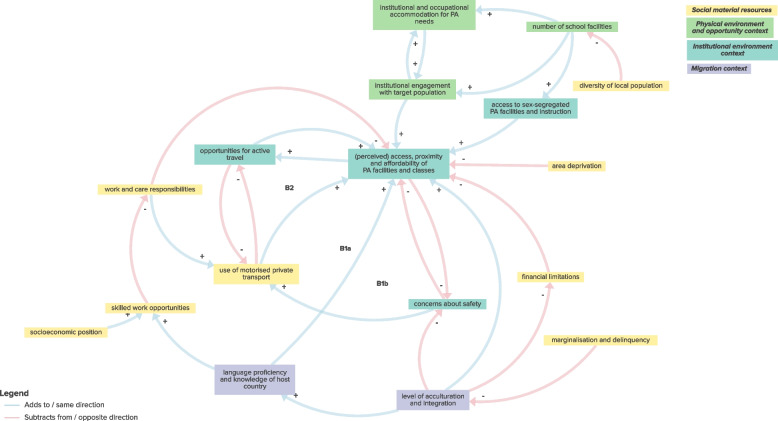
Sub-system CLD exploring interaction between environmental, migration and social and material resources factors

The sub-system CLD exploring the interaction between environmental, migration and social and material resources factors included 3 balancing feedback loops which supported an overarching hypothetical mechanism whereby multi-faceted access characteristics can sustain the perceived inaccessiblity of facilities or the use of motorised transport rather than active travel where facilities are perceived as being too distant (Fig. 6; Table 1; Supplementary File 3). More specifically, as illustrated, e.g., by balancing feedback loop B1b, PA facilities and classes that are perceived as inaccessible or far away are more likely to generate safety concerns related to reaching them, due to traffic or unfamiliar settings. These concerns increase the likelihood of using motorized private transport, such as cars, which reduces opportunities to incorporate active travel into daily routines. This, in turn, could otherwise enhance the perceived accessibility or proximity of local PA facilities and classes.

## Discussion

This study contributes an evidence-based conceptual systems model of how physical and institutional environments, migration context and individual factors interact to create a set of capabilities for physical activity in ethnic minority groups living in Europe. The conceptual model is supported by an established theoretical framework, the capabilities approach, and a comprehensive and systematic overview of evidence in the field, drawing on 41 studies. A total of 26 feedback loops were identified through causal loop diagramming, providing a preliminary indication that the emergent outcome of low levels of physical activity is supported by numerous reinforcing and balancing mechanisms. This pluralistic approach to literature selection permits insights into both how factors could shape persistently low levels of activity and how interventions or policies could interact with those factors to mitigate, and possibly reshape the direction of, their effect. Such insights make an important contribution to the field in moving beyond identifying potential relationships between isolated factors to indicating the ways in which these relationships are sustained and could be modified.

Taken together, the overarching hypothetical mechanisms of the 4 sub-systems suggest that a key influence on physical activity in ethnic minority groups living in Europe is the actual and perceived access to appropriate facilities, classes, instruction and advice. This is supported by previous evidence [30, 31]. Our findings suggest that perceived and actual access—determined by an interaction between institutional environment, physical environmental, sociocultural factors, health communication and social and material resources – can provide initial entry-points to physical activity participation, or support sustained physical activity, by alleviating other pressures on individuals. Over time, this participation in physical activity strengthens an individual’s motivation, capability and perceived opportunity to be active. However, if participation in physical activity is not facilitated through improved access, predominantly sociocultural mechanisms suppressing physical activity could be reinforced.

The CLDs suggest that the institutional environment and physical environment may both play an important role in providing actual and perceived access to facilities, classes, instruction and advice. For example, workplace physical activity classes, institutional engagement with the target population, concerns about safety of the physical environment and the provision of sex-segregated facilities or instruction were factors on feedback loops which were interpreted as underpinning overarching hypothetical mechanisms. Alongside an understanding of these environmental factors, the identified feedback loops support potential interaction with factors related to the migration context. This interaction highlights the importance of acknowledging unique factors such as language proficiency, knowledge of the host country and level of acculturation and integration which both shape the way ethnic minority groups experience institutional and physical environments and inform the influence of socioecological factors (such as knowledge, physical health, gender roles or use of private transport) on physical activity. As feedback loops are considered to drive the functioning of a system in producing emergent outcomes [19], it is important to note that contextual factors were implicated in all identified feedback loops.

The use of causal loop diagramming to explicate hypothetical mechanisms as reinforcing or balancing feedback loops is important as it helps us understand how physical activity, as an emergent outcome of a complex adaptive system, may be sustained by system functioning. This understanding lends further support to the suggestion that isolated interventions are unlikely to be effective if they only target one of many feedback loops [32]. Moreover, our delineation of complex adaptive processes permits insight into how wider system changes might be achieved over a longer time period leading to eventual changes in physical activity levels. For example, our results suggest that increasing physical activity through multi-component, multi-level interventions to increase perceived and actual access to physical activity opportunities (including access to appropriate facilities, instruction and advice) could mitigate the influence of sociocultural factors in the short-term. These opportunities could further stimulate physical activity through supportive psychosocial and sociocultural mechanisms (e.g. prioritisation of physical activity, opportunities for social connection), creating a potential ‘positive spiral’ which leads to increased physical activity levels in the longer term.

Our comprehensive overview of the determinants of physical activity, which we have interpreted as hypothetical mechanisms using systems dynamics theory and the capabilities approach, can inform the development, implementation and evaluation of multi-component, multi-level interventions and policies which aim to create the conditions to equitably promote physical activity across populations. Cross-sectoral and cross-disciplinary working, inclusive leadership and structural engagement between diverse partners is likely to be needed to simultaneously address the overarching mechanisms identified in this study [33, 34].

Identifying leverage points for intervention or policy was beyond the scope of this study. However, the sub-system CLDs can be used to hypothesise the potential knock-on effects of intervening at certain points in the system. Interventions in a system may address different levels of the system, with less impactful interventions aiming to change the value of individual factors within a system or modify a relationship between factors within a system, and more impactful interventions aiming to change the dynamics which underpin the goals and paradigm of a system which are responsible for driving emergent outcomes such as low levels of physical activity [19, 35].

Our identification of overarching hypothetical mechanisms is not only useful for understanding the structure and potential functioning of the system under study, but also for the development of interventions and policies which could attempt to target deeper levels of system change, such as the goals of the system. Simultaneous efforts to disrupt or strengthen the feedback loops supporting these mechanisms may enable sustained change in system functioning and reduce the possibility of interventions being suppressed by counteractive mechanisms (which may not be discernible without this comprehensive understanding of the system drawn from a wide range of evidence).

Differences in the numbers of factors and density of connections across the 4 sub-system CLDs could portend a limitation of using the evidence base to build our CLDs rather than participatory methods, such as group model building, which draws on individuals’ knowledge of a system. While our systematic search and selection strategy restricts potential bias in our utilisation of the evidence, it does mean that gaps in the evidence base will also appear as ‘gaps’ in the CLDs. It is therefore likely that the CLDs will lack specification of factors and connections for determinants that: have only been assessed in the general population (not explored in relation to ethnicity or race); are distal influences on physical activity; or have more often been examined in relation to ‘ultimate’ health outcomes rather than physical activity. Similarly, other factors related to minoritisation or discrimination, such as age, disability, low socioeconomic status, sexual orientation or pregnancy may be important for the pathways examined in this studied but were not included as the selected evidence limited our ability to adopt an intersectional perspective. Similarly, the predominance of certain study designs and methods in the evidence is likely to have shaped our CLDs: for example, 7 of the identified feedback loops were balancing loops, in part due to the inclusion of intervention studies in our literature review. Finally, it is probable that our CLDs reflect a lack of evidence for specific groups within our target population, owing to the difficulty of conducting research with new immigrants, undocumented immigrants, or those not registered with healthcare; our findings may be less generalisable to these groups.

It is necessary to interpret our findings in line with the objective of the study: to develop a conceptual systems-based model of the determinants of physical activity for ethnic minority groups in Europe. This model is based on predominantly qualitative cross-sectional studies which should not be used to provide evidence of causal relationships between factors, but do justify the mapping of hypothetical relationships. On the other hand, the qualitative nature of most studies also facilitated the identification of mechanisms underlying physical activity. Qualitative studies are arguably well suited to tease out longer, even non-linear pathways. This may have led to more granularity for the identified mechanisms. All assessed studies were deemed to be of satisfactory quality for inclusion. Otherwise, quality assessments were not integrated into our analysis or interpretation of our findings, which is a limitation of the current study. As a consequence, we were not able to specify the relative importance of the identified mechanisms. In future studies, resources could be dedicated to integrating quality assessments by using notation to demarcate the quality of evidence supporting each connection in a CLD (e.g. dashed versus solid connection arrows to demarcate lower versus higher quality evidence). Moreover, it is important to remember that mechanisms are discussed in terms of leading to outcomes at a group level rather than an individual level. There is a high degree of heterogeneity between and within the sub-groups studied in the evidence used to develop our CLDs (as discussed in [21, 22]), both in individuals’ characteristics such as age or access to resources and individuals’ exposure to or experience of contextual factors. We do not anticipate that mechanisms will be relevant or equally important across all individuals. For example, the component of “time and energy constraints arising from family and work commitments”, might be particularly applicable to adults with caring responsibilities, who might typically be younger, female and/or living in extended family groups. Finally, we have drawn on evidence from ethnic minority groups, not evidence comparing ethnic minority and ‘majority’ ethnic groups; it is not possible to know whether our hypothetical mechanisms generalise beyond our target population. Nevertheless, the results of this study in terms of specification of mechanisms underlying physical activity might increase the transferability of findings, more than when only evidence on specific determinants is available. These mechanisms might not only inform hypotheses as to whether these mechanisms can be expected in a specific context, but also will also help to understand why specific interventions might (not) work in different contexts.

In addition to incorporating new evidence as it is generated, future research could include physical activity outcomes in a systems-based conceptual model, to demonstrate the pathways from capabilities for physical activity to achieved functionings of being active. These pathways, and the pathways from contextual factors to capabilities, could be further delineated across different physical activity domains or ethnic minority groups. Adopting an intersectional lens when analysing these pathways would permit the research to inform the development of more sensitive interventions and policies [36–38].

Our conceptual model of how contextual and individual factors interact to create a set of capabilities for physical activity in ethnic minority groups living in Europe adds to other studies which have developed our understanding of the complex influences on physical activity [39–41]. If physical activity is understood to be an emergent outcome of a complex adaptive system, it is vital that this complexity is acknowledged in the development, implementation and evaluation of physical activity interventions and policies, in order to effectively tackle persistently low levels of physical activity in ethnic minority groups.

## Conclusions

This study contributes an evidence-based conceptual systems model which elucidates how low levels of physical activity in ethnic minority groups in Europe could be supported by reinforcing and balancing mechanisms involving factors relating to physical and institutional environments, migration context and individuals. A pluralistic approach to literature selection supports novel insights into how factors could interact to support persistently low levels of activity, moving beyond the identification of potential relationships between isolated factors to indicating the ways in which these relationships are sustained and could be modified by intervention or policy.

### Supplementary Information

Supplementary Material 1: Supplementary File 1. Search terms.

Supplementary Material 2: Supplementary File 2. Characteristics of included studies.

Supplementary Material 3: Supplementary File 3. Sub-system feedback loops presented individually.

## Data Availability

Data sharing not applicable to this article as no datasets were generated or analysed during the current study.

## Abbreviations

B: Balancing feedback loop
CLDs: Causal loop diagrams
PA: Physical activity
PEN: Policy Evaluation Network
R: Reinforcing feedback loop
