# Supplementary material for: Hypothetical mechanisms driving physical activity levels in ethnic minority groups living in Europe: a systematically identified evidence-based conceptual systems model
Source: Int J Behav Nutr Phys Act. 2024 Aug 7;21:87. doi: 10.1186/s12966-024-01626-2 (PMC11304888; doi:10.1186/s12966-024-01626-2)
Supplement: Supplementary file 1 — Supplementary Material 1: Supplementary File 1. Search terms. [file 12966_2024_1626_MOESM1_ESM.pdf]

Supplementary File 1: Search terms

Web of science

# 10	#9 AND #8 AND #3<br>Indexes=SCI-EXPANDED, SSCI, A&HCI
# 9	#6 OR #5<br>Indexes=SCI-EXPANDED, SSCI, A&HCI
# 8	#7 OR #4 OR #2<br>Indexes=SCI-EXPANDED, SSCI, A&HCI
# 7	**TOPIC:** (pala* or panam*or papua new guinea or paragu* or per* or phillipen* or rwand* or samo* or sao tome* and principe* or Senegal* or Seychelles or sierra leon* or solomon islands or Somali* or south afric* or south suda* or sri lank* or st luc* or st vincen* or suda* or surina* or Swaziland or Syrian arab republic or tajikista* or tanzan* or thail* or "timoe leste*" or tog* or tong* or turkmensista* or tuval* or ugand* or uzbekist* or vanuat* or venezuel* or vietna* or west bank and gaza or yemen* or zambi* or zimbabw*.ti,ab.)<br>Indexes=SCI-EXPANDED, SSCI, A&HCI
# 6	**TOPIC:** (*"physical activit*"/ or *"motor activit*"/ or excerci*.mp. or movement.mp. or or lifestyle*.mp. or behavio*.mp. or inactivt*.mp. or walk*.ti.ab.)<br>Indexes=SCI-EXPANDED, SSCI, A&HCI
# 5	**TOPIC:** (*"Motor Activity"/ or "Walking".mp. or "Movement".mp. / or *"physical activit*")<br>Indexes=SCI-EXPANDED, SSCI, A&HCI
# 4	**TOPIC:** (((((((*chinese*/ or *china/ or *irish traveller*/ or gyps*.mp. or *roma/ or *Asian/ or *Pakistan*/ or *banglades*/ or *turk*/ or *iran/ or *Iranian*/ or *irak*/ or *Maghreb*/ or *tunisi*/ or *morocco*/ or *liby*/ or *algeri*/ or *Surinam*/ or *latin American*/ or *cameroo*/ or *equado*/ or *eritre*/ or *chil*/ or *estoni*/ or *latvi*/ or *lithuani*/ or *goergi*/ or *armeni*/ or *azerbaija*/ or *kazakhsta*/ or *ukrain*/ or *belaru*/ or *moldov*/ or *croati*/ or *Czech republic*/ or *pol*/ or *romani*/ or *serbi*/ or *slovaki*/ or *sloveni*/ or *albani*/ or *bulgari*/ or *macedoni/ or *montenegr*/ or *angol*/ or *beliz*/ or *beni*/ or *bhuta*/ or *boliv*/ or *botswan*/ or *brazil*/ or *Burkina faso/ or *burund*/ or *chad/ or *colombi*/ or *cong*/ or *costa ric*/ or *ivoria*/ or *comor*/ or *cub*/ or *White/) and Black Caribbean.mp.) or *white/) and Black African.mp.) or *White/) and Asian.mp.) or *Mixed ethnic background/ or *multiple ethnic background*/ or *Central African Republic/ or djibout*.mp. or domini*.mp. or equado*.mp. or egyp*.mp. or ethiop*.mp. or fij*.mp. or gabo*.mp. or gambit*.mp. or ghan*.mp. or grenad*.mp. or guatemal*.mp. or guine*.mp. or guinea Bissau.mp. or guyan*.mp. or hait*.mp. or hondura*.mp. or hungar*.mp. or indones*.mp. or ira*.mp. or jamaic*.mp. or jord*.mp. or keny*.mp. or kiribat*.mp. or kore*.mp. or koso*.mp. or leban*.mp. or lesot*.mp. or liber*.mp. or macedon*.mp. or madagassc*.mp. or malaw*.mp. or lao pdr.mp. or malays*.mp. or mald*.mp. or marshall island*.mp. or maurit*.mp. or mexic*.mp. or micronesi*.mp. or moldov*.mp. or mongol*.mp. or mozambi*.mp. or myanma*.mp. or namib*.mp. or nep*.mp. or nicaragu*.mp. or nig*.mp. or nigeri*.mp. or Dominican republic.mp. or el-salvado*.mp. or kyrgyz republic.ti,ab.)

Indexes=SCI-EXPANDED, SSCI, A&HCI

\# 3 **TOPIC:** (Europe/ or *Russia/ or *Ukraine/ or *France/ or *Spain/ or *Sweden/ or *Norway/ or *Germany/ or *Finland/ or *Poland/ or *Italy/ or *Great Britain/ or *Romania/ or *"Republic of Belarus"/ or *Kazakhstan/ or *Greece/ or *Bulgaria/ or *Iceland/ or *Hungary/ or *Portugal/ or *Austria/ or *Czech Republic/ or *Serbia/ or *Ireland/ or *Latvia/ or *Bosnia-Herzegovina/ or *Croatia/ or *Lithuania/ or *Slovakia/ or *Estonia/ or *Denmark/ or *Netherlands/ or *Switzerland/ or *Moldova/ or *Belgium/ or *Albania/ or *"Macedonia (Republic)"/ or *Turkey/ or *Slovenia/ or *Montenegro/ or *cyprus/ or *malta/ or *Azerbaijan/ or *Luxembourg/ or *Georgia/ or *Andorra/ or *Liechtenstein/ or *Monaco/ or *Vatican City/ or *San Marino/ or (Europ or Russia or Ukraine or France or Spain or Sweden or Norway or Germany or (Finland or Poland or Italy or United Kingdom or Great Britain or Romania or Belarus) or (Kazakhstan or Greece or Bulgaria or Iceland or Hungary or Portugal or Austria or Czech Republic or Serbia or Republic of Ireland or Latvia) or (Lithuania or Croatia or Slovakia or Estonia or Denmark or Netherlands or Switzerland or Moldova or Belgium or Albania or Macedonia or Turkey or Slovenia or Montenegro or Cyprus or Azerbaijan or Luxembourg or Georgia or Andorra or Malta or Liechtenstein or San Marino or Monaco or Vatican city) or (Bosnia adj1 Herzegovina)).ti,ab.)
Indexes=SCI-EXPANDED, SSCI, A&HCI

\# 2 **TOPIC:** (*"Emigrants and Immigrants" or *"cultural diversity" or *"Minority Groups" or *"Transients and Migrants" or *"Ethnic Groups" or multiculturalism or "ethnic minorit*" or BME or "black minorit*" or ethnic* or asylum seeker* or refugee* or "african caribbean*" or *West Indies or *Afro-caribbean* or *Non-white or Coloured population or Black* or Afric* or Indi* or Caucasian* or Caribbean* or Arab* or "Black Afric*" or "South Asia*" or "Trinidad and Tobago" or emigrant* or immigrant* or minorit* or migrant* or minorit*)
Indexes=SCI-EXPANDED, SSCI, A&HCI

\# 1 **TOPIC:** (*"Emigrants and Immigrants" or *"cultural diversity" or *"Minority Groups" or *"Transients and Migrants" or *"Ethnic Groups" or multiculturalism or "ethnic minorit*" or BME or "black minorit*" or ethnic* or asylum seeker* or refugee* or "african caribbean*" or *West Indies or *Afro-caribbean* or *Non-white or Coloured population or Black* or Afric* or Indi* or Caucasian* or Caribbean* or Arab* or "Black Afric*" or "South Asia*" or "Trinidad and Tobago" or emigrant* or immigrant* or minorit* or migrant* or minorit*)
Indexes=SCI-EXPANDED, SSCI, A&HCI

Searcing articles and titles/abstracts only

\# 9 #8 AND #6 AND #2
Indexes=SCI-EXPANDED, SSCI, A&HCI

\# 8 #7 OR #1
Indexes=SCI-EXPANDED, SSCI, A&HCI

# 7 (TS=((*"Motor Activity"/ or "Walking" or "Movement" or *"physical activit*").ti,ab.)) *AND* **DOCUMENT TYPES:** (Article)
Indexes=SCI-EXPANDED, SSCI, A&HCI

# 6 #5 OR #4 OR #3
Indexes=SCI-EXPANDED, SSCI, A&HCI

# 5 (TS=(pala* or panam*or papua new guinea or paragu* or per* or phillipen* or rwand* or samo* or sao tome* and principe* or Senegal* or Seychelles or sierra leon* or solomon islands or Somali* or south afric* or south suda* or sri lank* or st luc* or st vincen* or suda* or surina* or Swaziland or Syrian arab republic or tajikista* or tanzan* or thail* or "timoe leste*" or tog* or tong* or turkmensista* or tuval* or ugand* or uzbekist* or vanuat* or venezuel* or vietna* or west bank and gaza or yemen* or zambi* or zimbabw*.ti,ab.)) *AND* **DOCUMENT TYPES:** (Article)
Indexes=SCI-EXPANDED, SSCI, A&HCI

# 4 (TS=((*"Emigrants and Immigrants" or *"cultural diversity" or *"Minority Groups" or *"Transients and Migrants" or *"Ethnic Groups" or multiculturalism or "ethnic minorit*" or BME or "black minorit*" or ethnic* or asylum seeker* or refugee* or "african caribbean*" or *West Indies or *Afro-caribbean* or *Non-white or Coloured population or Black* or Afric* or Indi* or Caucasian* or Caribbean* or Arab* or "Black Afric*" or "South Asia*" or "Trinidad and Tobago" or emigrant* or immigrant* or minorit* or migrant* or minorit*).ti,ab.)) *AND* **DOCUMENT TYPES:** (Article)
Indexes=SCI-EXPANDED, SSCI, A&HCI

# 3 (TS=(((((((*chinese*/ or *china/ or *irish traveller*/ or gyps*.mp. or *roma/ or *Asian/ or *Pakistan*/ or *banglades*/ or *turk*/ or *iran/ or *Iranian*/ or *irak*/ or *Maghreb*/ or *tunisi*/ or *morocco*/ or *liby*/ or *algeri*/ or *Surinam*/ or *latin American*/ or *cameroo*/ or *equado*/ or *eritre*/ or *chil*/ or *estoni*/ or *latvi*/ or *lithuani*/ or *goergi*/ or *armeni*/ or *azerbaija*/ or *kazakhsta*/ or *ukrain*/ or *belaru*/ or *moldov*/ or *croati*/ or *Czech republic*/ or *pol*/ or *romani*/ or *serbi*/ or *slovaki*/ or *sloveni*/ or *albani*/ or *bulgari*/ or *macedoni/ or *montenegr*/ or *angol*/ or *beliz*/ or *beni*/ or *bhuta*/ or *boliv*/ or *botswan*/ or *brazil*/ or *Burkina faso/ or *burund*/ or *chad/ or *colombi*/ or *cong*/ or *costa ric*/ or *ivoria*/ or *comor*/ or *cub*/ or *White/) and Black Caribbean.mp.) or *white/) and Black African.mp.) or *White/) and Asian.mp.) or *Mixed ethnic background/ or *multiple ethnic background*/ or *Central African Republic/ or djibout*.mp. or domini*.mp. or equado*.mp. or egyp*.mp. or ethiop*.mp. or fij*.mp. or gabo*.mp. or gambit*.mp. or ghan*.mp. or grenad*.mp. or guatemal*.mp. or guine*.mp. or guinea Bissau.mp. or guyan*.mp. or hait*.mp. or hondura*.mp. or hungar*.mp. or indones*.mp. or ira*.mp. or jamaic*.mp. or jord*.mp. or keny*.mp. or kiribat*.mp. or kore*.mp. or koso*.mp. or leban*.mp. or lesot*.mp. or liber*.mp. or macedon*.mp. or madagassc*.mp. or malaw*.mp. or lao pdr.mp. or malays*.mp. or mald*.mp. or marshall island*.mp. or maurit*.mp. or mexic*.mp. or micronesi*.mp. or moldov*.mp. or mongol*.mp. or mozambi*.mp. or myanma*.mp. or namib*.mp. or nep*.mp. or nicaragu*.mp. or nig*.mp. or nigeri*.mp. or Dominican republic.mp. or el-salvado*.mp. or kyrgyz republic.ti,ab.)) *AND* **DOCUMENT TYPES:** (Article)
Indexes=SCI-EXPANDED, SSCI, A&HCI

# 2 (TS=(Europe/ or *Russia/ or *Ukraine/ or *France/ or *Spain/ or *Sweden/ or *Norway/ or *Germany/ or *Finland/ or *Poland/ or *Italy/ or *Great Britain/ or *Romania/ or *"Republic of Belarus"/ or *Kazakhstan/ or *Greece/ or *Bulgaria/ or *Iceland/ or *Hungary/ or *Portugal/ or *Austria/ or *Czech Republic/ or *Serbia/ or *Ireland/ or *Latvia/ or *Bosnia-Herzegovina/ or *Croatia/ or *Lithuania/ or *Slovakia/ or *Estonia/ or *Denmark/ or *Netherlands/ or *Switzerland/ or *Moldova/ or *Belgium/ or *Albania/ or *"Macedonia (Republic)"/ or *Turkey/ or *Slovenia/ or *Montenegro/ or *cyprus/ or *malta/ or *Azerbaijan/ or *Luxembourg/ or *Georgia/ or *Andorra/ or *Liechtenstein/ or *Monaco/ or *Vatican City/ or *San Marino/ or (Europ or Russia or Ukraine or France or Spain or Sweden or Norway or Germany or (Finland or Poland or Italy or United Kingdom or Great Britain or Romania or Belarus) or (Kazakhstan or Greece or Bulgaria or Iceland or Hungary or Portugal or Austria or Czech Republic or Serbia or Republic of Ireland or Latvia) or (Lithuania or Croatia or Slovakia or Estonia or Denmark or Netherlands or Switzerland or Moldova or Belgium or Albania or Macedonia or Turkey or Slovenia or Montenegro or Cyprus or Azerbaijan or Luxembourg or Georgia or Andorra or Malta or Liechtenstein or San Marino or Monaco or Vatican city) or (Bosnia adj1 Herzegovina)).ti,ab.)) *AND* **DOCUMENT TYPES:** (Article)
Indexes=SCI-EXPANDED, SSCI, A&HCI

# 1 (TS=(*"physical activit*"/ or *"motor activit*"/ or excerci*.mp. or movement.mp. or lifestyle*.mp. or behavio*.mp. or inactivt*.mp. or walk*.ti,ab.)) *AND* **DOCUMENT TYPES:** (Article)
Indexes=SCI-EXPANDED, SSCI, A&HCI

OVID

16	("Emigrants and Immigrants" or "cultural diversity" or "Minority Groups" or "Transients and Migrants" or "Ethnic Groups" or multiculturalism or "ethnic minorit*" or BME or "black minorit*" or ethnic* or asylum seeker* or refugee* or "african caribbean*" or West Indies or Afro-caribbean* or Non-white or Coloured population or Black* or Afric* or Indi* or Caucasian* or Caribbean* or Arab* or "Black Afric*" or "South Asia*" or "Trinidad and Tobago" or emigrant* or immigrant* or minorit* or migrant* or minorit*).kw,ti.
17	("Motor Activity" or "Walking" or "Movement" or "physical activity").kw,ti.
18	(((((((chinese* or china or irish traveller* or gyps* or roma or Asian or Pakistan* or banglades* or turk* or iran or Iranian* or irak* or Maghreb* or tunisi* or morocco* or liby* or algeri* or Surinam* or latin American* or cameroo* or equado* or eritre* or chil* or estoni* or latvi* or lithuani* or goergi* or armeni* or azerbaija* or kazakhsta* or ukrain* or belaru* or moldov* or croati* or Czech republic* or pol* or romani* or serbi* or slovaki* or sloveni* or albani* or bulgari* or macedoni or montenegr* or angol* or beliz* or beni* or bhuta* or boliv* or botswan* or brazil* or Burkina faso or burund* or chad or colombi* or cong* or costa ric* or ivoria* or comor* or cub* or White) and Black Caribbean) or white) and Black African) or White) and Asian) or "Mixed ethnic background" or "multiple ethnic background" or Central African Republic or djibout* or domini* or equado* or egyp* or ethiop* or fij* or gabo* or gambit* or ghan* or grenad* or guatemal* or guine* or guinea Bissau or guyan* or hait* or hondura* or hungar* or indones* or ira* or jamaic* or jord* or keny* or kiribat* or kore* or koso* or leban* or lesot* or liber* or macedon* or madagassc* or malaw* or lao or malays* or mald* or marshall island* or maurit* or mexic* or micronesi* or moldov* or mongol* or mozambi* or myanma* or namib* or nep* or nicaragu* or nig* or nigeri* or Dominican republic or el-salvado* or kyrgyz republic).kw,ti.
19	(Europe or Russia or Finland or Poland or Italy or Great Britain or Romania or "Republic of Belarus" or Kazakhstan or Greece or Bulgaria or Iceland or Hungary or Portugal or Austria or Czech Republic or Serbia or Ireland or Latvia or Bosnia-Herzegovina or Croatia or Lithuania or Slovakia or Estonia or Denmark or Netherlands or Switzerland or Moldova or Belgium or Albania or "Macedonia (Republic)" or Malta or Ukraine or France or Spain or Sweden or Norway or Germany or United Kingdom or Belarus or Republic of Ireland or Latvia or Lithuania or Croatia or Slovakia or Estonia or Denmark or Netherlands or Switzerland or Moldova or Belgium or Albania or Macedonia or Turkey or Slovenia or Montenegro or Cyprus or Azerbaijan or Luxembourg or Georgia or Andorra or Malta or Liechtenstein or San Marino or Monaco or Vatican city or Bosnia Herzegovina).kw,ti.
20	(((((pala* or panam*or papua new guinea or paragu* or per* or phillipen* or rwand* or samo* or sao tome) and principe) or Senegal* or Seychelles or sierra leon* or solomon islands or Somali* or south afric* or south suda* or sri lank* or st luc* or st vincen* or suda* or surina* or Swaziland or Syrian arab republic or tajikista* or tanzan* or thail* or timoe-leste or tog* or tong* or turkmensista* or tuval* or ugand* or uzbekist* or vanuat* or venezuel* or vietna* or west bank) and gaza) or yemen or zambi* or zimbabw*).kw,ti.
21	(physical* or activit* or motor activit* or excerci* or movement or inactivt* or walk*).kw,ti.
22	17 or 21
23	16 or 18 or 20
24	19 and 22 and 23
25	24 and "human" [Subjects]

CINAHL

S5	Europe/ or *Russia/ or *Ukraine/ or *France/ or *Spain/ or *Sweden/ or *Norway/ or *Germany/ or *Finland/ or *Poland/ or *Italy/ or *Great Britain/ or *Romania/ or *"Republic of Belarus"/ or *Kazakhstan/ or *Greece/ or *Bulgaria/ or *Iceland/ or *Hungary/ or *Portugal/ or *Austria/ or *Czech Republic/ or *Serbia/ or *Ireland/ or *Latvia/ or *Bosnia-Herzegovina/ or *Croatia/ or *Lithuania/ or *Slovakia/ or *Estonia/ or *Denmark/ or *Netherlands/ or *Switzerland/ or *Moldova/ or *Belgium/ o ...
S4	((((pala* or panam* or papua new guinea or paragu* or per* or phillipen* or rwand* or samo* or sao tome) and principe) or Senegal* or Seychelles or sierra leon* or solomon islands or Somali* or south afric* or south suda* or sri lank* or st luc* or st vincen* or suda* or surina* or Swaziland or Syrian arab republic or tajikista* or tanzan* or thail* or timoe-leste or tog* or tong* or turkmensista* or tuval* or ugand* or uzbekist* or vanuat* or venezuel* or vietna* or west bank) and gaza) or yeme ...
S3	((((((*chinese*/ or *china/ or *irish traveller*/ or gyps*.mp. or *roma/ or *Asian/ or *Pakistan*/ or *banglades*/ or *turk*/ or *iran/ or *Iranian*/ or *irak*/ or *Maghreb*/ or *tunisi*/ or *morocco*/ or *liby*/ or *algeri*/ or *Surinam*/ or *latin American*/ or *cameroo*/ or *equado*/ or *eritre*/ or *chil*/ or *estoni*/ or *latvi*/ or *lithuani*/ or *goergi*/ or *armeni*/ or *azerbaija*/ or *kazakhsta*/ or *ukrain*/ or *belaru*/ or *moldov*/ or *croati*/ or *Czech republic*/ or *pol* ...
S2	*"Emigrants and Immigrants"/ or *cultural diversity/ or *Minority Groups/ or *"Transients and Migrants"/ or *Ethnic Groups/ or multiculturalism*.mp. or *ethnic minorit*/ or BME.mp. or black minorit*.mp. or ethnic*.mp. or asylum seeker*.mp. or refugee*.mp. or african caribbean*.mp. or *West Indies/ or *Afro-caribbean*/ or *Non-white/ or *Coloured population/ or *Black*/ or *Afric*/ or *Indi*/ or *Caucasian*/ or *Caribbean*/ or *Arab*/ or *Black Afric*/ or *South Asia*/ or *Trinidad/) an ...
S1	physical* or activit* or motor activit* or excerci* or movement or inactivt* or walk*
