# Supplementary material for: Hypothetical mechanisms driving physical activity levels in ethnic minority groups living in Europe: a systematically identified evidence-based conceptual systems model
Source: Int J Behav Nutr Phys Act. 2024 Aug 7;21:87. doi: 10.1186/s12966-024-01626-2 (PMC11304888; doi:10.1186/s12966-024-01626-2)
Supplement: Supplementary file 2 — Supplementary Material 2: Supplementary File 2. Characteristics of included studies. [file 12966_2024_1626_MOESM2_ESM.pdf]

Supplementary File 2: Characteristics of included studies

Table 1: Characteristics of included studies

| **First author, year** | **Title** | **Source review** | **Study design** | **Study population (ethnic minority group(s), sex)** | **Sample characteristics (age)** | **Number of participants** | **Setting, country** | **Recruitment** | **Physical activity domain** | **Physical activity measurement** | **CLD Ref** | **Quality** |
|---|---|---|---|---|---|---|---|---|---|---|---|---|
| Asamane, 2019 (1) | Perceptions and Factors Influencing Eating Behaviours and Physical Function in Community-Dwelling Ethnically Diverse Older Adults: A Longitudinal Qualitative Study | Sawyer | Longitudinal qualitative; semi-structured interviews | African, Indian, Pakistani, Bangladeshi or Caribbean adults | 60 years and older | 92 at baseline; 81 at follow-up | England, Birmingham area | Community leaders | Physical function | Self-reported participation in exercise, general fitness level, strength | 8 | 0.75 |
| Benn, 2013 (2) | Meeting needs of Muslim girls in school sport: case studies exploring cultural and religious diversity | Langøien | Case studies; qualitative | Muslim girls and stakeholders | 16-17 years; England: 8 state schools; Denmark: 1 class | England: 109 girls, 19 teachers, 32 parents; Denmark: 42 girls and boys | England; Demark | Representative sample | General PA, physical education | England: survey, focus groups, semi-structured interview; Denmark: survey, video observation of sport/PA lessons, interview | 9 | 0.45 |

| | | | | | | | | | | | | |
|---|---|---|---|---|---|---|---|---|---|---|---|---|
| Beune, 2010 (3) | Inhibitors and enablers of physical activity in multiethnic hypertensive patients: qualitative study | Langøien | Qualitative inductive | Ghanaian, African-Surinamese, or White Dutch adults | 35-65 years | 26 women, 20 men (16 Ghanaian; 15 African-Surinamese; 15 White Dutch) diagnosed with hypertension | Amsterdam, The Netherlands | Purposive sampling through health-care centres | General PA | Self-report | 11 | 0.8 |
| Bukman, 2017 (4) | Effectiveness of the MetSLIM lifestyle intervention targeting individuals of low socio-economic status and different ethnic origins with elevated waist-to-height ratio | Sawyer | Lifestyle intervention; quantitative | Turkish; Moroccan; or Dutch adults | 30-70 years | 124 women; 25 men (71 Turkish; 6 Moroccan; 59 Dutch) | Socioeconomically deprived neighbourhoods, The Netherlands | General Practitioners; community centres | General PA | Self-report | 12 | 0.79 |

| | | | | | | | | | | | | |
|---|---|---|---|---|---|---|---|---|---|---|---|---|
| Dagkas, 2006 (5) | Young Muslim women's experience of Islam and physical education in Greece and Britain: a comparative study | Langøien | Interpretive study; qualitative | Greek Turkish girls; British Asian women | Greek Turkish girls: 13-15 years; British Asian women: 18-21 years | Greek Turkish girls: 24 at school; British Asian women: 20 at university | Greece, UK | British group participating in existing project | General PA, physical education, sport | Semi-structured interview | 14 | 1.0 |
| Dagkas, 2011 (6) | Multiple voices: improving participation of Muslim girls in physical education and school sport | Langøien | Case studies; qualitative | Muslim girls | 5-16 years | 109 girls; 19 teachers; 32 parents; additional focus groups with 36 girls | West Midlands, England | Representative sample of schools | General PA, physical education, school sport | Focus groups; questionnaire | 15 | 0.95 |
| Emadian, 2017 (7) | A Mixed-Methods Examination of Physical Activity and Sedentary Time in Overweight and Obese South Asian Men Living in the United Kingdom | Sawyer | Cross-sectional; mixed methods; semi-structured interviews; questionnaire | Indian; Pakistani; and Bangladeshi men | 18-65 years | 54 men (PA assessment); sub-group of 31 men (semi-structured interviews) | Greater London, England | Mosques, temples and community centres | Total MVPA | Accelerometer; self-report | 21 | 0.5 |
| Frahsa, 2020 (8) | Capabilities for Physical Activity by Turkish- and Russian-Speaking Immigrants Aged 65 Years and Older in | Sawyer | Qualitative; group interviews | Turkish- and Russian-speaking adults with migration history | 65 years and older | 12 women; 7 men | Germany | Purposive snowball sampling | General PA | Questionnaire | 23 | 0.9 |

| | | | | | | | | | | | | |
|---|---|---|---|---|---|---|---|---|---|---|---|---|
| | Germany: A Qualitative Study | | | | | | | | | | | |
| Fullagar, 2020 (9) | Action research with parkrun UK volunteer organizers to develop inclusive strategies | Sawyer | Action research; mixed-methods | Target population of action research: adults with ethnic and religious backgrounds; survey: White adults (93,1%) | Mean age 41,9 (survey) | Action research: parkrun volunteers; survey: 309 men, 332 women | United Kingdom | Parkrun volunteers | Sport club membership | Questionnaire; interviews | 24 | 0.59 |
| Gele, 2015 (10) | Beyond Culture and Language: Access to Diabetes Preventive Health Services among Somali Women in Norway | Sawyer | Qualitative; unstructured interviews | Somalian women | 25 years and older | 30 | Oslo, Norway | Snowball sampling | General PA | n/a | 27 | 0.65 |

| | | | | | | | | | | | | |
|---|---|---|---|---|---|---|---|---|---|---|---|---|
| Hayes, 2002 (11) | Patterns of physical activity and relationship with risk markers for cardiovascular disease and diabetes in Indian, Pakistani, Bangladeshi and European adults in a UK population | Langøien | Cross-sectional; quantitative | European; Indian; Pakistani; and Bangladeshi adults | 25-75 years | 825 Europeans; 684 Indian, Pakistani or Bangladeshi | Newcastle upon Tyne, England | Random selection | General PA | Questionnaire | 29 | 0.82 |
| Hornby-Turner, 2014 (12) | A comparison of physical activity and sedentary behaviour in 9-11 year old British Pakistani and White British girls: a mixed methods study | Langøien | Cross-sectional; mixed methods (PA determinants only from qualitative interviews) | British-Pakistani; and White British girls | 9-11 years | 75 British Pakistani; 70 White British; parents of 19 girls | North East England | Participation letter sent to parents in 8 schools | General PA, Vigorous PA, sports, outdoor play, active travel to school | Questionnaire, PA interview, accelerometer | 32 | 0.65 |

| | | | | | | | | | | | |
|---|---|---|---|---|---|---|---|---|---|---|---|
| Horne, 2010 (13) | The influence of primary health care professionals in encouraging exercise and physical activity uptake among White and South Asian older adults: experiences of young older adults | Langøien | Ethnographic approach; qualitative | South-Asian and White British adults | 60-70 years | Focus groups: 87; interviews: 40 | North West England | Purposive sampling from fieldwork observations in leisure groups and social centres | General PA, exercise | Self-report, focus groups, in-depth interviews | 33 | 0.8 |
| Horne, 2012 (14) | Attitudes and beliefs to the uptake and maintenance of physical activity among community-dwelling South Asians aged 60-70 years: a qualitative study | Langøien | Ethnographic approach; qualitative | South Asian adults | 60-70 years | Focus groups: 29; interviews: 17 | United Kingdom | Purposive sampling from fieldwork observations in leisure groups and social centres | General PA | Self-report, focus group, in-depth interviews | 34 | 0.7 |
| Horne, 2013 (15) | Perceived barriers to initiating and maintaining physical activity among South Asian and White British adults in their 60s living in the United | Langøien | Exploratory qualitative approach | South-Asian (first generation migrants) and White British adults | 60-70 years | Focus groups: 70 women, 47 men; interviews: 34 women, 23 men | North West England | Purposive sample | General PA, exercise | Focus groups, in-depth interviews | 35 | 0.8 |

| | | | | | | | | | | | | |
|---|---|---|---|---|---|---|---|---|---|---|---|---|
| | Kingdom: a qualitative study | | | | | | | | | | | |
| Hosper, 2008 (16) | Motivational factors mediating the association between acculturation and participation in sport among young Turkish and Moroccan women in the Netherlands | Langøien | Cross-sectional; quantitative | Turkish and Moroccan women | 15-30 years | 258 Turkish women; 170 Moroccan women | Amsterdam, The Netherlands | Random sample from population register | General PA, sport, leisure-time PA | Questionnaire | 37 | 1.0 |
| Jonsson, 2013 (17) | Is acculturation associated with physical activity among female immigrants in Sweden? | Langøien | Cross-sectional; quantitative | Women living in Sweden: born in Finland; Chile; and Iraq | 18-65 years | 1651 | Stockholm and Botkyrka, Sweden | Random sample from population register | Leisure-time PA | Postal questionnaire | 41 | 1.0 |
| Jorgensdotter, 2018 (18) | Cultural Background and Societal Influence on Coping Strategies for Physical Activity Among Immigrant Women | Sawyer | Qualitative; focus groups | Women with first generation migration history (14 countries) | 35-60 years | 22 | Gothenburg, Sweden | Primary care | General PA | n/a | 42 | 0.95 |

| | | | | | | | | | | | | |
|---|---|---|---|---|---|---|---|---|---|---|---|---|
| Kay, 2006 (19) | Daughters of Islam: Family influences on Muslim young women's participation in sport | Langøien | Interviews; qualitative | Muslim girls (Bangladeshi; black African; Arab descent) and their families | 13-18 years | 7 women; 6 families | Midlands town, UK | Participation in Widening Access Through Sport project | General PA, sport | Interviews | 43 | 0.55 |
| Khunti, 2007 (20) | Primary prevention of type-2 diabetes and heart disease: action research in secondary schools serving an ethnically diverse UK population | Langøien | Cross-sectional; mixed methods, reporting on qualitative | 5 schools | 11-15 years | 3601 | Leicester, England | Representative sample through schools | General PA | Questionnaire | 44 | 0.5 |
| Lawton, 2006 (21) | 'I can't do any serious exercise': barriers to physical activity amongst people of Pakistani and Indian origin with Type 2 diabetes | Langøien | In-depth interviews; qualitative | Pakistani and Indian patients (first and second generation migrants) diagnosed with Type 2 diabetes | Over 18 years | Pakistani: 12 women, 11 men; Indian: 5 women, 4 men | Edinburgh, Scotland | Clinical (general practitioner) and local community recruitment, purposive and snowball sampling | General PA | In-depth interviews | 51 | 0.75 |

| | | | | | | | | | | | | |
|---|---|---|---|---|---|---|---|---|---|---|---|---|
| Lenneis, 2016a (22) | Health, physical activity and the body: an inquiry into the lives of female migrant cleaners in Denmark | Sawyer | Semi-structured interviews; qualitative | Female cleaners with a non-Western background (Turkey; Pakistan; China; India; Macedonia; Kosovo; Morocco; Palestine; The Philippines; Serbia; Tanzania; a majority of participants were Muslim) | 27-62 years | 33 | Denmark | Previous participants in workplace exercise programme | Recreational PA | Face-to-face semi-structured interviews | 53 | 0.9 |
| Lenneis 2016b (23) | Playing after work? Opportunities and challenges of a physical activity programme for female cleaners | Sawyer | Qualitative; semi-structured interviews | Female cleaners with first-generation migration history (Turkey; China; India; Kosovo; Macedonia; Morocco; Serbia; 2 participants from Denmark; a majority of the participants were Muslim) | 27-62 years | 25 | Copenhagen, Denmark | Cleaners at university or hospital | Participation in culturally-sensitive workplace PA intervention | Interviews and observation | 54 | 0.85 |

| | | | | | | | | | | | | |
|---|---|---|---|---|---|---|---|---|---|---|---|---|
| Lenneis, 2017a (24) | Too tired for exercise? The work and leisure of female cleaners in Denmark | Sawyer | Qualitative | Female cleaners with first-generation migration history (Turkey; China; India; Kosovo; Macedonia; Morocco; Palestine; The Philippines; Serbia; Tanzania; a majority self-identified as Muslim) | 27-62 years | 25 | Copenhagen, Denmark | Cleaners at university or hospital, previous participants in workplace PA programme | Leisure-time PA | Semi-structured interviews | 55 | 0.8 |
| Lenneis, 2017b (25) | When girls have no opportunities and women have neither time nor energy: the participation of Muslim female cleaners in recreational physical activity | Sawyer | Qualitative | Female cleaners with first-generation migration history (Turkey; Pakistan; China; India; Kosovo; Macedonia; Morocco; Palestine; The Philippines; Serbia; Tanzania; a majority self-identified as Muslim) | 27-50 years | 26 0.85 | Copenhagen, Denmark | Cleaners at university or hospital; previous participants in workplace PA programme | Leisure-time PA | Semi-structured interviews; observation | 56 | 0.85 |

| | | | | | | | | | | | | |
|---|---|---|---|---|---|---|---|---|---|---|---|---|
| Lenneis, 2020 (26) | Women-only swimming as a space of belonging | Sawyer | Qualitative | Muslim women with a migration background | 26-58 years | 14 | Aarhus, Denmark | Swimmers at women-only swimming sessions; sports club management | Swimming; provision of women-only swimming sessions | Ethnographic field observation; semi-structured interviews | 57 | 0.9 |
| Marconnot, 2019 (27) | Recognition of barriers to physical activity promotion in immigrant children in Spain: A qualitative case study | Sawyer | Case study; qualitative (unstructured and semi-structured interviews, focus groups) | Target population: 10-14 year olds with migrant background; participants: parents, teachers, school principal, community leaders | Target population: 10-14 year olds; participants: mean age 43,4 years | 9 women, 16 men | Alcorón, Spain | Educational centres, Catholic parishes; purposive sampling | Barriers to PA promotion | n/a | 61 | 0.95 |
| Molaodi, 2012 (28) | Neighbourhood food and physical activity environments in England, UK: does ethnic density matter? | Langøien | Environment – modelling | White British; Black African; Black Caribbean; Indian; Pakistani; Bangladeshi; Chinese; and Irish | Deprived areas; all age groups | n/a | United Kingdom | n/a | Physical activity facilities | Lists from Sport England | 64 | 1.0 |

| | | | | | | | | | | | | |
|---|---|---|---|---|---|---|---|---|---|---|---|---|
| Nicolaou, 2012 (29) | Influences on body weight of female Moroccan migrants in the Netherlands: a qualitative study | Langøien | Focus groups; qualitative | Moroccan women | 51 | Amsterdam: 22; Morocco: 29 | Amsterdam, The Netherlands; Morocco | Amsterdam: mother-child and women's centres; Morocco: Al Hoceima town, small village, medium-sized village | General PA | Focus groups | 67 | 0.8 |
| Nicolaou, 2014 (30) | Development of a diabetes prevention program for Surinamese South Asians in the Netherlands | Sawyer | Intervention development; qualitative | Surinamese South Asian adults | Adults | 5 focus groups with 4-6 participants; interviews with 6 participants | The Hague and Amsterdam, The Netherlands | General Practitioner records; South Asian associations | MVPA | n/a | 68 | 0.8 |
| Oosterwerff, 2014 (31) | Effect of vitamin D supplementation on physical performance and activity in non-western immigrants | Sawyer | RCT; quantitative | Adults with non-Western migration history (predominantly: Moroccan; Surinamese; and Turkish) with vitamin D deficiency | 20 - 65 years | 78 women, 52 men | The Netherlands | General Practitioners, mosques, community centres and health centres | Total PA; exercise capacity | Accelerometer, questionnaire | 72 | 0.96 |

| | | | | | | | | | | | | |
|---|---|---|---|---|---|---|---|---|---|---|---|---|
| Pallan, 2012 (32) | Contextual influences on the development of obesity in children: a case study of UK South Asian communities | Langøien | Focus groups with stakeholders; qualitative | Target population: children with Indian, Pakistani or Bangladeshi migration background; study participants: adult stakeholders | Target population: primary school-aged children | 60 females, 8 males | United Kingdom | 8 communities with predominantly South Asian pupils, serving disadvantaged populations | General PA | Focus groups | 74 | 0.8 |
| Persson, 2014 (33) | Somali women's view of physical activity - a focus group study | Sawyer | Focus group; qualitative | Somali women living in Sweden | 17 to 67 years | 26 | Sweden | Snowball methods | General PA | Focus groups | 76 | 0.9 |
| Reimers, 2019 (34) | Are there disparities in different domains of physical activity between school-aged migrant and non-migrant children and adolescents? Insights from Germany | Sawyer | Quantitative | Children without German nationality or with parents with migration history | 6-17 years | 3,323 children; 210 with two-sided migration background, 230 with one-sided migration background, 2883 with non-migration background | Germany | German Health and Examination Survey for Children and Adolescents | Sport club participation, outdoor play, active travel to school, extra-curricular PA, PA outside of sports clubs | Questionnaire | 81 | 1.0 |

| | | | | | | | | | | | | |
|---|---|---|---|---|---|---|---|---|---|---|---|---|
| Samkange-Zeeb, 2015 (35) | Assessing the Acceptability and Usability of an Internet-Based Intelligent Health Assistant Developed for Use among Turkish Migrants: Results of a Study Conducted in Bremen, Germany | Sawyer | Intervention development; participatory research design | Children and adults with Turkish migration background | 11-70 years | 28 females, 13 males | Bremen, Germany | Family and youth centres, community networks | Development of internet-based PA assistant | n/a | 85 | 0.65 |
| Sandstrom, 2015 (36) | Attitudes to and Experiences of Physical Activity among Migrant Women from Former Yugoslavia | Sawyer | Qualitative; semi-structured interviews | Bosnian women living in Sweden | Born in 1953-1990 | 7 | Malmo, Sweden | Purposive snowball sampling | General PA | Interview | 86 | 0.85 |
| Sodergren, 2008 (37) | Arranging appropriate activities immigrant women's ideas of enabling exercise | Langøien | Explorative qualitative | Women with migration history from Chile; Iraq; and Turkey | 26-65 years | 63 | Stockholm, Sweden | Multi-recruitment strategy (associations, centres, education facilities) | General PA, exercise | Focus groups | 93 | 0.75 |

| | | | | | | | | | | | |
|---|---|---|---|---|---|---|---|---|---|---|---|
| Sriskantharajah, 2007 (38) | Promoting physical activity among South Asian women with coronary heart disease and diabetes: what might help? | Langøien | Explorative qualitative | South Asian women (Indian; Pakistani; Bangladeshi; East African; and Sri Lankan) with coronary heart disease or Type 2 diabetes | 26-70 years | 15 | United Kingdom | 3 general practices, purposive sampling | General PA, exercise | Semi-structured interview | 96 | 0.9 |
| Steinbach, 2011 (39) | Cycling and the city: a case study of how gendered, ethnic and class identities can shape healthy transport choices | Langøien | In-depth interviews, fieldwork; qualitative | Workplaces with employees with White; Black; and Asian ethnicity | 18-64 years | 67 females, 11 males | London, United Kingdom | Purposive sampling (workplaces, volunteer group, cycle training schemes) | Cycling for transport | In-depth interviews, fieldwork | 97 | 0.8 |
| Walseth, 2006 (40) | Young Muslim women and sport: the impact of identity work | Langøien | Life-history; qualitative | Muslim women with second-generation migration history (Pakistan; Turkey; Morocco; Iran; Syria; Somalia; Gambia; Macedonia; and Kosovo) | 16-25 years | 21 women | Norway | Alumni of an elementary school in Oslo, sports clubs | General PA, sport | Life-story interviews | 103 | 1.0 |

| | | | | | | | | | | | | |
|---|---|---|---|---|---|---|---|---|---|---|---|---|
| Walseth, 2016 (41) | Sport within Muslim organizations in Norway: Ethnic segregated activities as arena for integration | Sawyer | Qualitative | Representatives of Muslim organisations; Bosnian; Somali; and Pakistani men | All ages | 7 | Oslo, Norway | Muslim organisations | Sports offer in mosques; sports participation | Semi-structured interviews, participant observation | 104 | 0.85 |

References


1. Asamane EA, Greig CA, Aunger JA, Thompson JL. Perceptions and Factors Influencing Eating Behaviours and Physical Function in Community-Dwelling Ethnically Diverse Older Adults: A Longitudinal Qualitative Study. Nutrients. 2019;11(6).
2. Benn T, Pfister G. Meeting needs of Muslim girls in school sport: Case studies exploring cultural and religious diversity. European Journal of Sport Science. 2013;13(5):567-74.
3. Beune EJ, Haafkens JA, Agyemang C, Bindels PJ. Inhibitors and enablers of physical activity in multiethnic hypertensive patients: qualitative study. J Hum Hypertens. 2010;24(4):280-90.
4. Bukman AJ, Teuscher D, Meershoek A, Renes RJ, van Baak MA, Feskens EJ. Effectiveness of the MetSLIM lifestyle intervention targeting individuals of low socio-economic status and different ethnic origins with elevated waist-to-height ratio. Public Health Nutr. 2017;20(14):2617-28.
5. Dagkas S, Benn T. Young Muslim women's experiences of Islam and physical education in Greece and Britain: a comparative study. Sport, Education and Society. 2006;11(1):21-38.
6. Dagkas S, Benn T, Jawad H. Multiple voices: improving participation of Muslim girls in physical education and school sport. Sport, Education and Society. 2011;16(2):223-39.
7. Emadian A, Thompson J. A Mixed-Methods Examination of Physical Activity and Sedentary Time in Overweight and Obese South Asian Men Living in the United Kingdom. Int J Environ Res Public Health. 2017;14(4).
8. Frahsa A, Streber A, Wolff AR, Rütten A. Capabilities for Physical Activity by Turkish- and Russian-Speaking Immigrants Aged 65 Years and Older in Germany: A Qualitative Study. J Aging Phys Act. 2020;28(4):567-79.
9. Fullagar S, Petris S, Sargent J, Allen S, Akhtar M, Ozakinci G. Action research with parkrun UK volunteer organizers to develop inclusive strategies. Health Promot Int. 2020;35(5):1199-209.
10. Gele AA, Torheim LE, Pettersen KS, Kumar B. Beyond Culture and Language: Access to Diabetes Preventive Health Services among Somali Women in Norway. J Diabetes Res. 2015;2015:549795.
11. Hayes L, White M, Unwin N, Bhopal R, Fischbacher C, Harland J, et al. Patterns of physical activity and relationship with risk markers for cardiovascular disease and diabetes in Indian, Pakistani, Bangladeshi and European adults in a UK population. J Public Health Med. 2002;24(3):170-8.

12. Hornby-Turner YC, Hampshire KR, Pollard TM. A comparison of physical activity and sedentary behaviour in 9–11 year old British Pakistani and White British girls: a mixed methods study. International Journal of Behavioral Nutrition and Physical Activity. 2014;11(1):74.
13. Horne M, Skelton D, Speed S, Todd C. The influence of primary health care professionals in encouraging exercise and physical activity uptake among White and South Asian older adults: experiences of young older adults. Patient Educ Couns. 2010;78(1):97-103.
14. Horne M, Skelton DA, Speed S, Todd C. Attitudes and beliefs to the uptake and maintenance of physical activity among community-dwelling South Asians aged 60-70 years: a qualitative study. Public Health. 2012;126(5):417-23.
15. Horne M, Skelton DA, Speed S, Todd C. Perceived barriers to initiating and maintaining physical activity among South Asian and White British adults in their 60s living in the United Kingdom: a qualitative study. Ethn Health. 2013;18(6):626-45.
16. Hosper K, Nierkens V, van Valkengoed I, Stronks K. Motivational factors mediating the association between acculturation and participation in sport among young Turkish and Moroccan women in the Netherlands. Prev Med. 2008;47(1):95-100.
17. Jönsson LS, Palmér K, Ohlsson H, Sundquist J, Sundquist K. Is acculturation associated with physical activity among female immigrants in Sweden? J Public Health (Oxf). 2013;35(2):270-7.
18. Jörgensdotter Wegnelius C, Petersson EL. Cultural Background and Societal Influence on Coping Strategies for Physical Activity Among Immigrant Women. J Transcult Nurs. 2018;29(1):54-63.
19. Kay T. Daughters of Islam:Family Influences on Muslim Young Women's Participation in Sport. International Review for the Sociology of Sport. 2006;41(3-4):357-73.
20. Khunti K, Stone MA, Bankart J, Sinfield P, Pancholi A, Walker S, et al. Primary prevention of type-2 diabetes and heart disease: action research in secondary schools serving an ethnically diverse UK population. J Public Health (Oxf). 2008;30(1):30-7.
21. Lawton J, Ahmad N, Hanna L, Douglas M, Hallowell N. 'I can't do any serious exercise': barriers to physical activity amongst people of Pakistani and Indian origin with Type 2 diabetes. Health Educ Res. 2006;21(1):43-54.
22. Lenneis V, Pfister G. Health, physical activity and the body: an inquiry into the lives of female migrant cleaners in Denmark. International Journal of Sport Policy and Politics. 2016;8(4):647-62.
23. Lenneis V, Pfister G. Playing after work? Opportunities and challenges of a physical activity programme for female cleaners. International Sports Studies. 2016;38(1):5-23.
24. Lenneis V, Pfister G. Too tired for exercise? The work and leisure of female cleaners in Denmark. Leisure Studies. 2017;36(4):530-41.
25. Lenneis V, Pfister G. When girls have no opportunities and women have neither time nor energy: the participation of Muslim female cleaners in recreational physical activity. Sport in Society. 2017;20(9):1203-22.
26. Lenneis V, Agergaard S, Evans AB. Women-only swimming as a space of belonging. Qualitative Research in Sport, Exercise and Health. 2020;14(1):37-52.
27. Marconnot R, Marín-Rojas AL, Delfa-de-la-Morena JM, Pérez-Corrales J, Gueita-Rodríguez J, Fernández-de-Las-Peñas C, et al. Recognition of Barriers to Physical Activity Promotion in Immigrant Children in Spain: A Qualitative Case Study. Int J Environ Res Public Health. 2019;16(3).
28. Molaodi OR, Leyland AH, Ellaway A, Kearns A, Harding S. Neighbourhood food and physical activity environments in England, UK: does ethnic density matter? Int J Behav Nutr Phys Act. 2012;9:75.

29. Nicolaou M, Benjelloun S, Stronks K, van Dam RM, Seidell JC, Doak CM. Influences on body weight of female Moroccan migrants in the Netherlands: A qualitative study. Health & Place. 2012;18(4):883-91.
30. Nicolaou M, Vlaar E, van Valkengoed I, Middelkoop B, Stronks K, Nierkens V. Development of a diabetes prevention program for Surinamese South Asians in the Netherlands. Health Promot Int. 2014;29(4):680-91.
31. Oosterwerff MM, Meijnen R, Schoor NM, Knol DL, Kramer MH, Poppel MN, et al. Effect of vitamin D supplementation on physical performance and activity in non-western immigrants. Endocr Connect. 2014;3(4):224-32.
32. Pallan M, Parry J, Adab P. Contextual influences on the development of obesity in children: a case study of UK South Asian communities. Prev Med. 2012;54(3-4):205-11.
33. Persson G, Mahmud AJ, Hansson EE, Strandberg EL. Somali women's view of physical activity--a focus group study. BMC Womens Health. 2014;14:129.
34. Reimers AK, Brzoska P, Niessner C, Schmidt SCE, Worth A, Woll A. Are there disparities in different domains of physical activity between school-aged migrant and non-migrant children and adolescents? Insights from Germany. PLoS One. 2019;14(3):e0214022.
35. Samkange-Zeeb F, Ernst SA, Klein-Ellinghaus F, Brand T, Reeske-Behrens A, Plumbaum T, et al. Assessing the Acceptability and Usability of an Internet-Based Intelligent Health Assistant Developed for Use among Turkish Migrants: Results of a Study Conducted in Bremen, Germany. Int J Environ Res Public Health. 2015;12(12):15339-51.
36. Sandström E, Bolmsjö I, Janzon E. Attitudes to and Experiences of Physical Activity among Migrant Women from Former Yugoslavia: -- a qualitative interview study about physical activity and its beneficial effect on heart health, in Malmö, Sweden. AIMS Public Health. 2015;2(2):194-209.
37. Södergren M, Hylander I, Törnkvist L, Sundquist J, Sundquist K. Arranging Appropriate Activities: Immigrant Women's Ideas of Enabling Exercise. Women's Health Issues. 2008;18(5):413-22.
38. Sriskantharajah J, Kai J. Promoting physical activity among South Asian women with coronary heart disease and diabetes: what might help? Fam Pract. 2007;24(1):71-6.
39. Steinbach R, Green J, Datta J, Edwards P. Cycling and the city: A case study of how gendered, ethnic and class identities can shape healthy transport choices. Social Science & Medicine. 2011;72(7):1123-30.
40. Walseth K. Young Muslim Women and Sport: the Impact of Identity Work. Leisure Studies. 2006;25(1):75-94.
41. Walseth K. Sport within Muslim organizations in Norway: ethnic segregated activities as arena for integration. Leisure Studies. 2016;35(1):78-99.
