# Supplementary material for: Hypothetical mechanisms driving physical activity levels in ethnic minority groups living in Europe: a systematically identified evidence-based conceptual systems model
Source: Int J Behav Nutr Phys Act. 2024 Aug 7;21:87. doi: 10.1186/s12966-024-01626-2 (PMC11304888; doi:10.1186/s12966-024-01626-2)
Supplement: Supplementary file 3 — Supplementary Material 3: Supplementary File 3. Sub-system feedback loops presented individually. [file 12966_2024_1626_MOESM3_ESM.pdf]

Supplementary File 3: Sub-system feedback loops presented individually

Environmental, migration and psychosocial factors sub-system

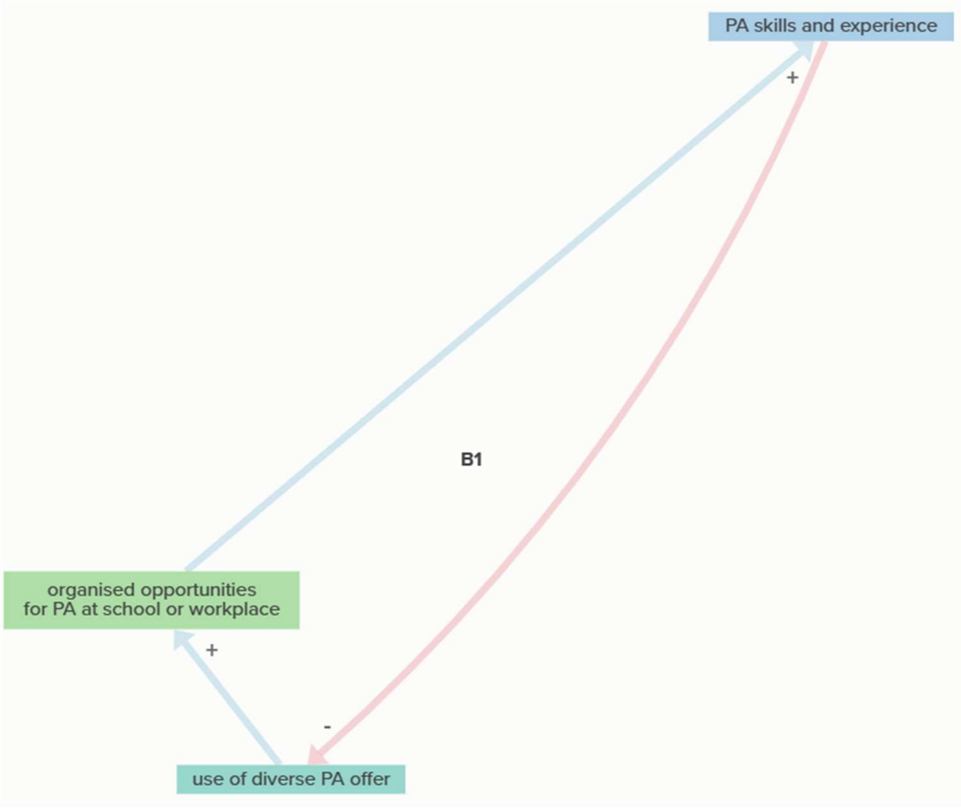

Balancing loop B1: *Bolstering PA skills and experience through organised PA opportunities*: Lower levels of PA skills and experience increases the need for a diverse PA offer, which can be served by taking up more organised opportunities for PA in school or workplace settings, increasing PA skills and experience.

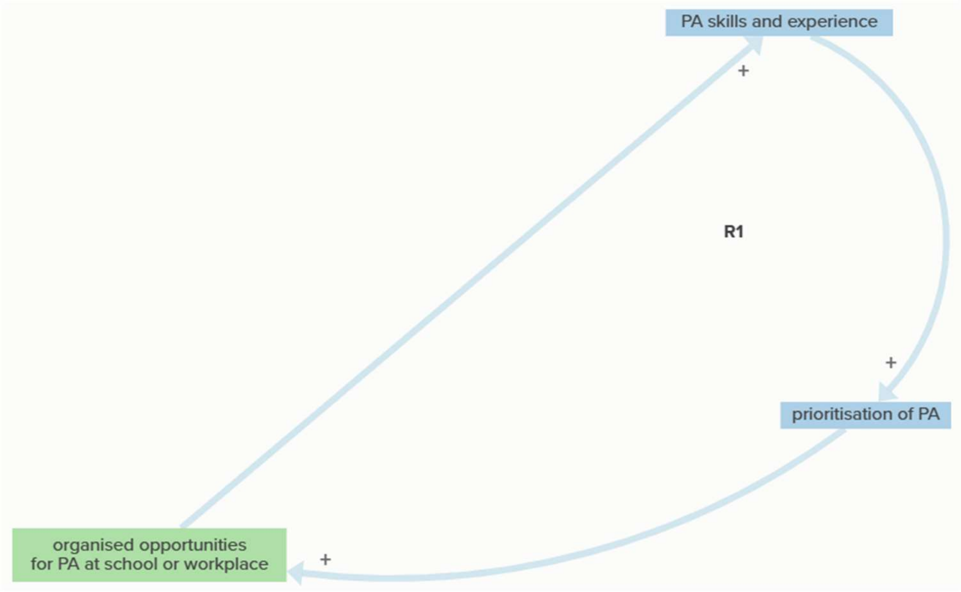

Reinforcing loop R1: *Prioritisation of PA leading to increased PA skills and experience:* Higher levels of PA skills and experience can lead to an individual prioritising PA and being more likely to take up organised PA opportunities, further improving their skills and experience.

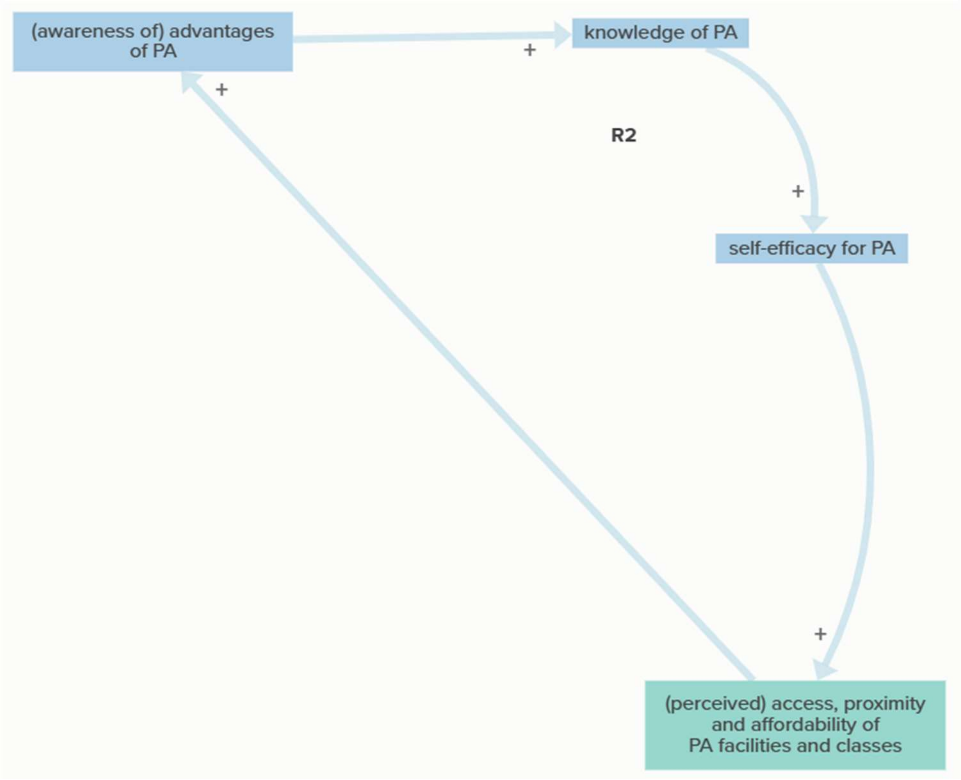

Reinforcing loop R2: *Bolstering awareness of PA advantages through increased access to PA facilities and classes:* Increased awareness of PA advantages can lead to increased knowledge of PA concepts and guidelines for health-promoting PA, which can increase self-efficacy for PA and make PA facilities and classes appear more welcoming and geographically accessible (e.g. by active transportation). Higher levels of attendance at PA facilities and classes can tacitly and explicitly increase awareness of PA advantages.

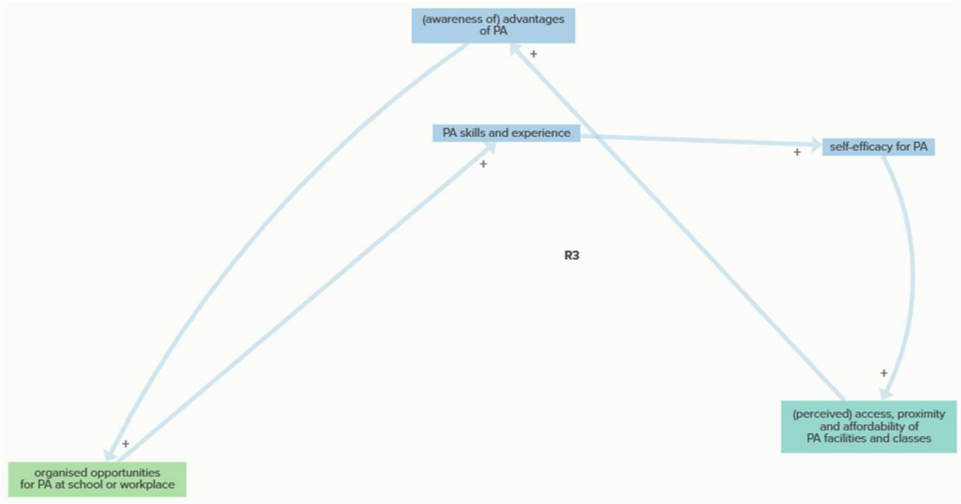

Reinforcing loop R3: *Strengthening PA skills and experience through increased PA participation*: A higher level of PA skills and experience increases an individual's self-efficacy for PA, which increases perceived access and proximity to PA facilities and classes (as they appear more welcoming and more accessible via active transport). Access to PA facilities and classes can tacitly and explicitly increase awareness of the advantages of PA (by observing the advantages, or being told about advantages via instruction), which encourages participation in organised PA opportunities at school or the workplace, further strengthening PA skills and experience.

Environmental, migration and sociocultural factors sub-system

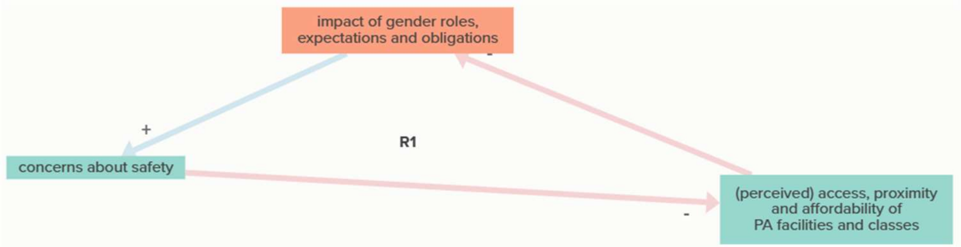

Reinforcing loop R1: *Concerns about safety leading to growing impact of gender roles, expectations and obligations through increasingly reduced access to PA facilities and classes:* Greater impact of gender roles, expectations and obligations lead to increased concerns about safety which can reduce perceived access or proximity to PA facilities and classes, which are deemed unsafe to

attend or reach. Reduced access to facilities and classes can be restrictive and frame such facilities or classes as unsuitable for girls and women, further increasing the impact of gender roles, expectations and obligations.

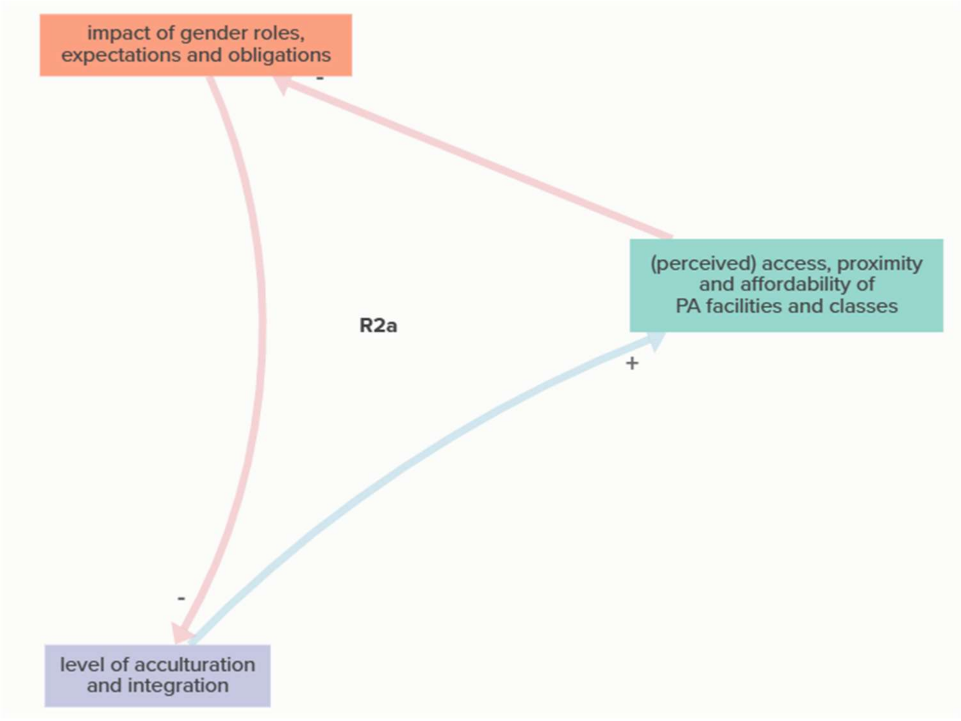

Reinforcing loop R2a: *Low levels of acculturation and integration leading to growing impact of gender roles, expectations and obligations through increasingly reduced access to PA facilities and classes.* Greater impact of gender roles, expectations and obligations can lead to lower levels of acculturation and integration in other communities, which could restrict access to PA facilities and classes as these are not signposted by other members of the community or are deemed unsuitable or unwelcoming. Reduced access to facilities and classes can be restrictive and frame such facilities or classes as unsuitable for girls and women, further increasing the impact of gender roles, expectations and obligations.

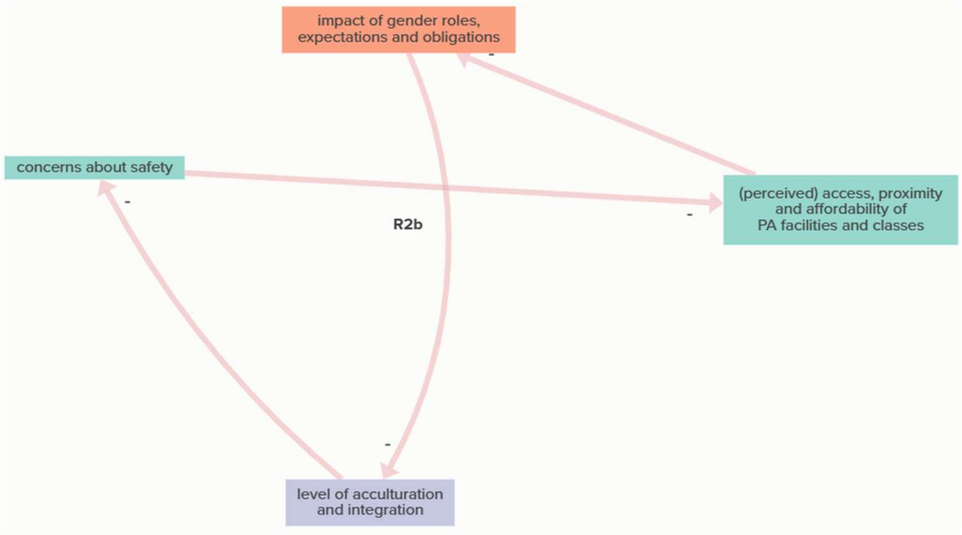

Reinforcing loop R2b: *Low levels of acculturation and integration leading to growing impact of gender roles, expectations and obligations through increasingly reduced access to PA facilities and classes and concerns about safety.* Greater impact of gender roles, expectations and obligations can lead to lower levels of acculturation and integration in other communities, which could increase concerns about safety in the local area, restricting access to local PA facilities and classes. Reduced access to facilities and classes can be restrictive and frame such facilities or classes as unsuitable for girls and women, further increasing the impact of gender roles, expectations and obligations.

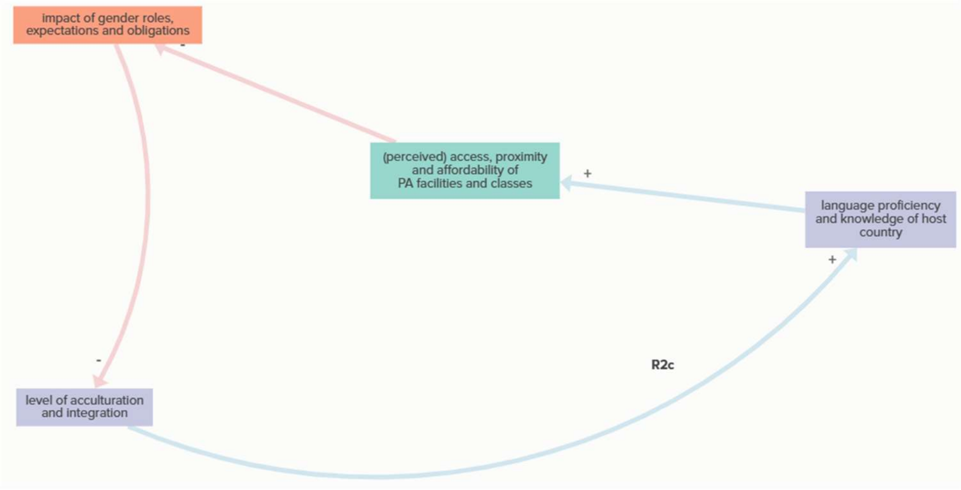

Reinforcing loop R2c: *Low levels of acculturation and integration leading to growing impact of gender roles, expectations and obligations through increasingly reduced access to PA facilities and classes and low language proficiency and knowledge of host country.* Greater impact of gender roles, expectations and obligations can lead to lower levels of acculturation and integration in other communities, which can restrict proficiency of the dominant language and knowledge of the host county. Lower levels of language proficiency and country knowledge can limit access to local and affordable PA facilities and classes as these are not signposted or have access rules around membership which are available to the individual. Reduced access to facilities and classes can be restrictive and frame such facilities or classes as unsuitable for girls and women, further increasing the impact of gender roles, expectations and obligations.

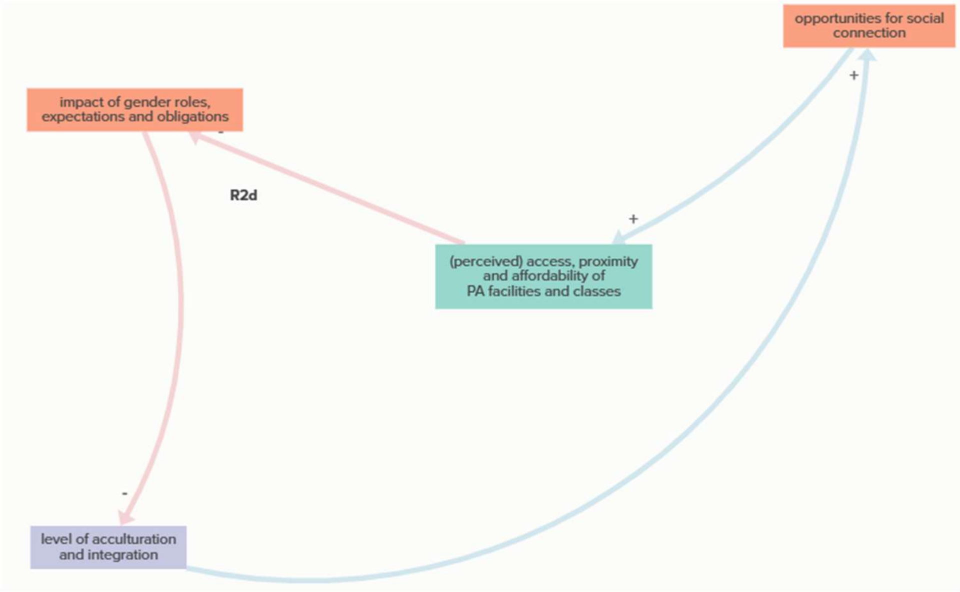

Reinforcing loop R2d: *Low levels of acculturation and integration leading to growing impact of gender roles, expectations and obligations through increasingly reduced access to PA facilities and classes and reduced opportunities for social connection.* Greater impact of gender roles, expectations and obligations can lead to lower levels of acculturation and integration in other communities, which can reduce opportunities for social connection with members of other groups in the community. This can lead to reduced access to PA facilities and classes as these can be unfamiliar and are not signposted by other members of the community. Reduced access to facilities and classes can be restrictive and frame such facilities or classes as unsuitable for girls and women, further increasing the impact of gender roles, expectations and obligations.

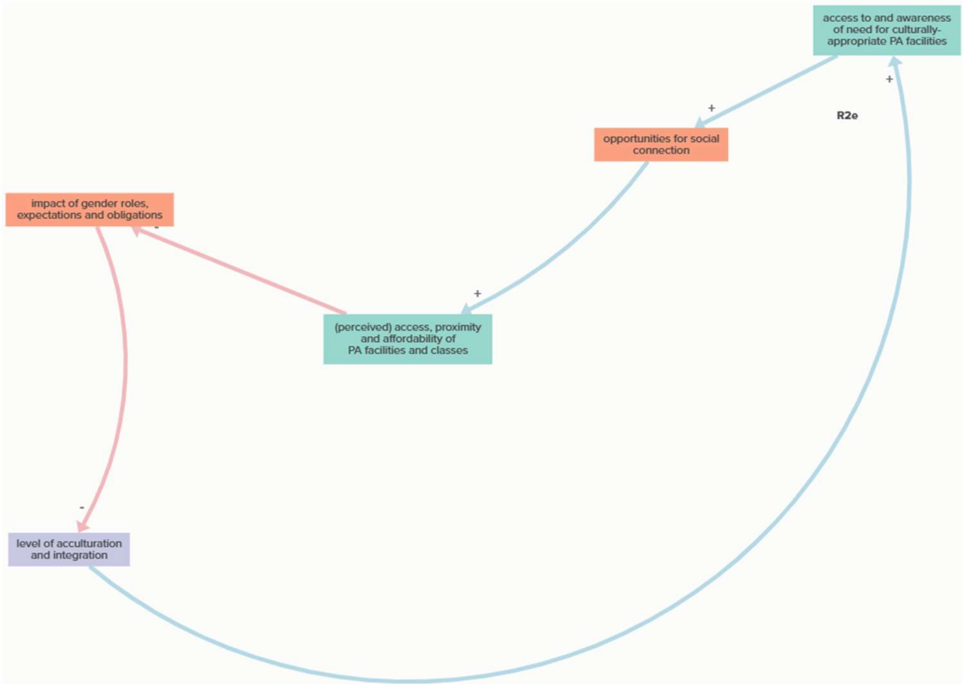

Reinforcing loop R2e: *Low levels of acculturation and integration leading to growing impact of gender roles, expectations and obligations through increasingly reduced access to PA facilities and classes and reduced opportunities for social connection.* Greater impact of gender roles, expectations and obligations can lead to lower levels of acculturation and integration in other communities, which could restrict access to and awareness of culturally-appropriate PA facilities as these are not signposted by other members of the community or are deemed unsuitable or unwelcoming. Reduced PA participation at such facilities reduces opportunities for social connection with other participants, which can further reduce access to general PA facilities as details of these facilities are not shared through social networks. Reduced access to facilities and classes can be restrictive and frame such facilities or classes as unsuitable for girls and women, further increasing the impact of gender roles, expectations and obligations.

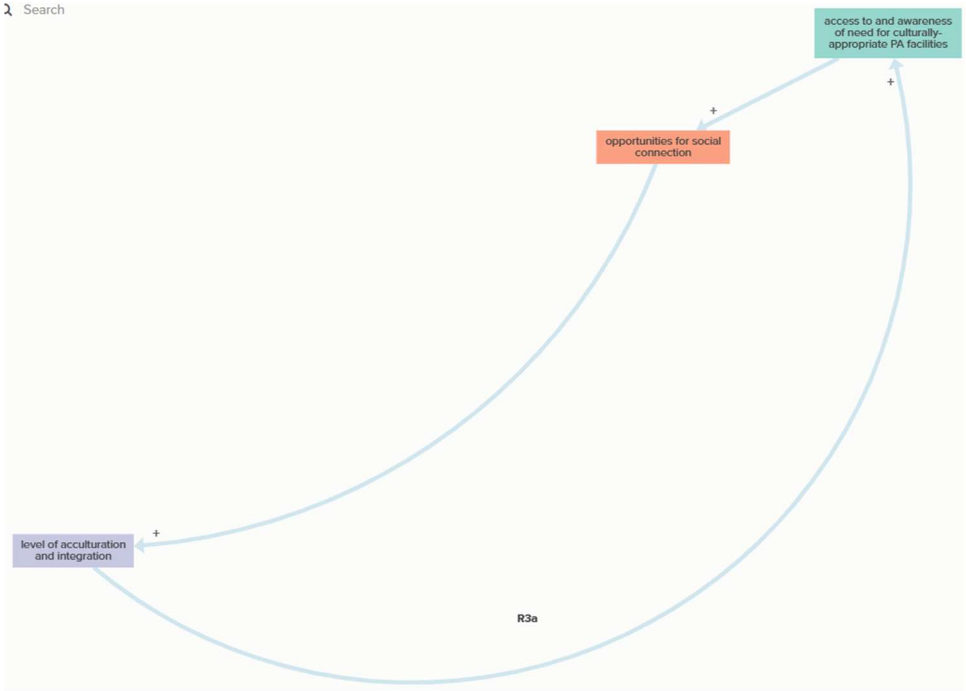

Reinforcing loop R3a: *High levels of acculturation and integration bolstered by opportunities for social connection obtained through increased access to culturally-appropriate physical activity facilities.* Greater levels of acculturation and integration can promote access to and awareness of culturally-appropriate PA facilities, as the community caters to the needs of the local population or existing facilities are signposted and seen as suitable and welcoming. Access to these facilities creates more opportunities for social connection within and between group members, further increasing an individual's level of acculturation and integration.

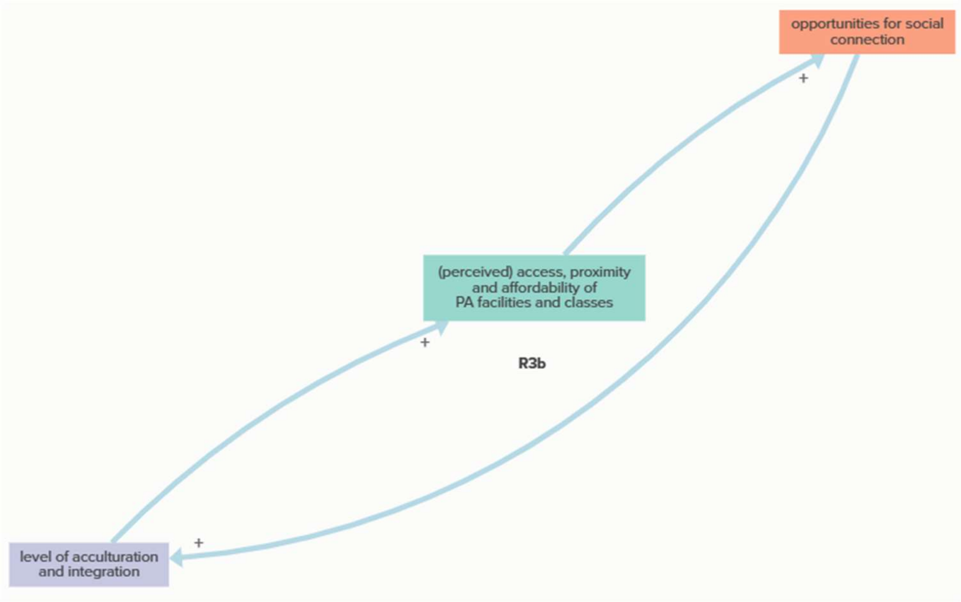

Reinforcing loop R3b: *High levels of acculturation and integration bolstered by opportunities for social connection obtained through increased access to physical activity facilities.* Greater levels of acculturation and integration can promote access to PA facilities and classes in general, as the community caters to the needs of the local population or existing facilities are signposted and seen as suitable and welcoming. Access to these facilities creates more opportunities for social connection within and between group members, further increasing an individual's level of acculturation and integration.

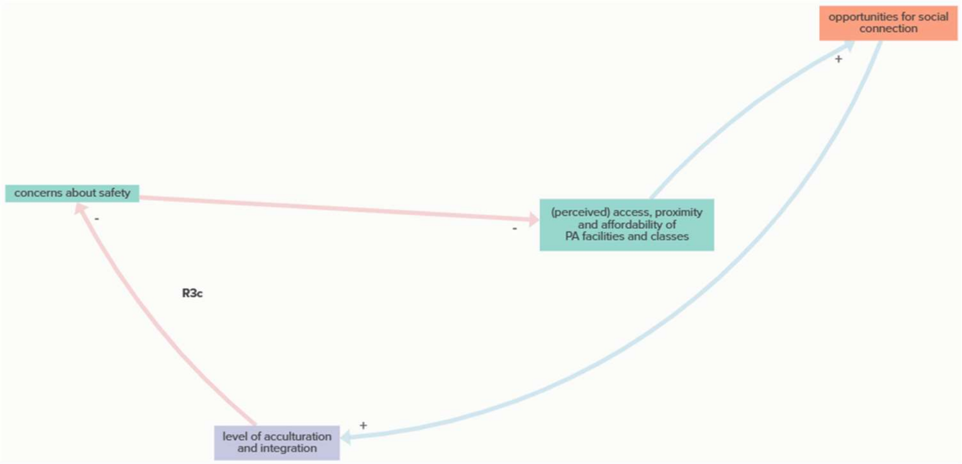

Reinforcing loop R3c: *High levels of acculturation and integration bolstered by opportunities for social connection obtained through increased access to physical activity facilities and classes which is facilitated by reduced concerns about safety.* Greater levels of acculturation and integration can reduce concerns about safety in the local area, making local PA facilities and classes appear more welcoming and safe. Use of such facilities increases opportunities for social connection with other users, building a social network that contributes to higher levels of integration.

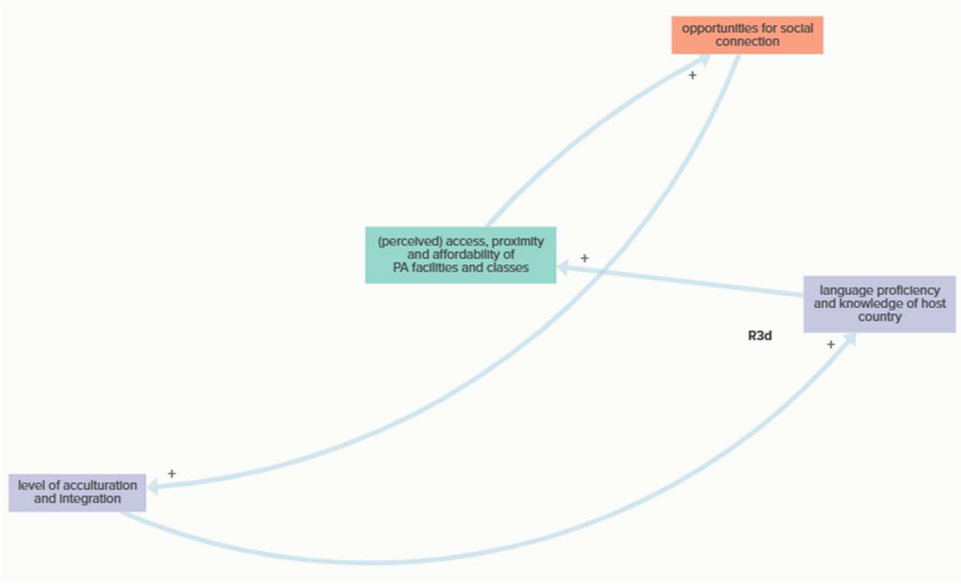

Reinforcing loop R3d: *High levels of acculturation and integration bolstered by opportunities for social connection obtained through increased access to physical activity facilities and classes which is facilitated by increased language proficiency and knowledge of the host country.* Greater levels of acculturation and integration can facilitate acquisition of the dominant language and increase knowledge of the host country, which can enable access to facilities and classes, with joining information or instruction in the dominant language. Access to these facilities creates more opportunities for social connection within and between group members, further increasing an individual's level of acculturation and integration.

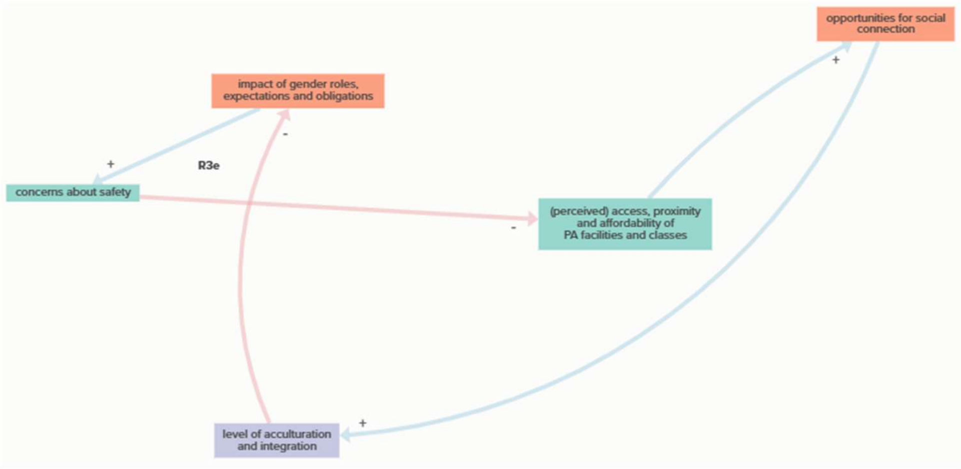

Reinforcing loop R3e: *High levels of acculturation and integration bolstered by opportunities for social connection obtained through increased access to physical activity facilities and classes which is facilitated by increased language proficiency and knowledge of the host country.* High levels of acculturation and integration can reduce the impact of gender roles, expectations and obligations for some groups, reducing concerns about safety for women and girls. Fewer concerns may increase access to PA facilities and classes, as they are deemed safe, increasing opportunities for social connection within and between group members, further increasing an individual's integration within their own and other groups.

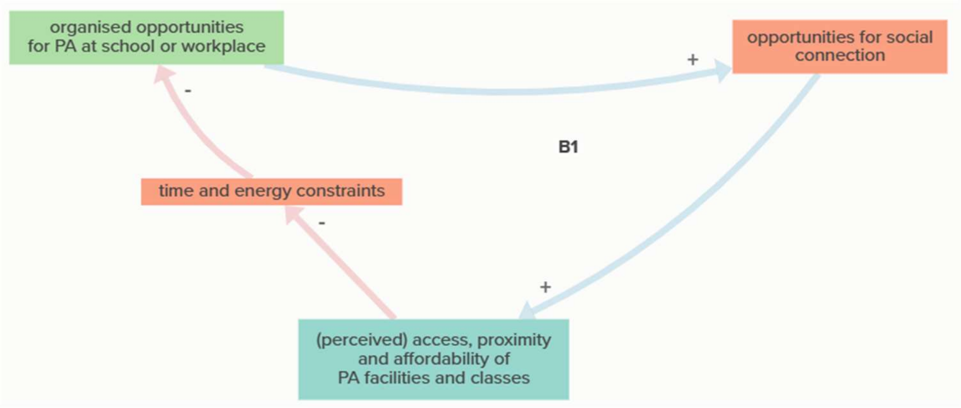

Balancing loop B1: *Time and energy constraints (arising from family and work commitments alongside additional challenges of living in a new country or facing discrimination) reduce participation in workplace physical activity classes and social connection which could otherwise increase access to local physical activity facilities and classes.* Greater time and energy constraints experienced by individuals with family and work commitments alongside additional challenges of living in a new country or facing discrimination can limit participation in organised PA opportunities in the school or workplace. In turn, missing such opportunities limits opportunities for social connection in those settings, limiting access to PA facilities and classes which may have been shared through that social network. However, accessing such facilities or classes, locally or further afield, are reported to increase time and energy constraints further, therefore, not accessing these facilities protects against further demands on an individual's pressured time and energy resources.

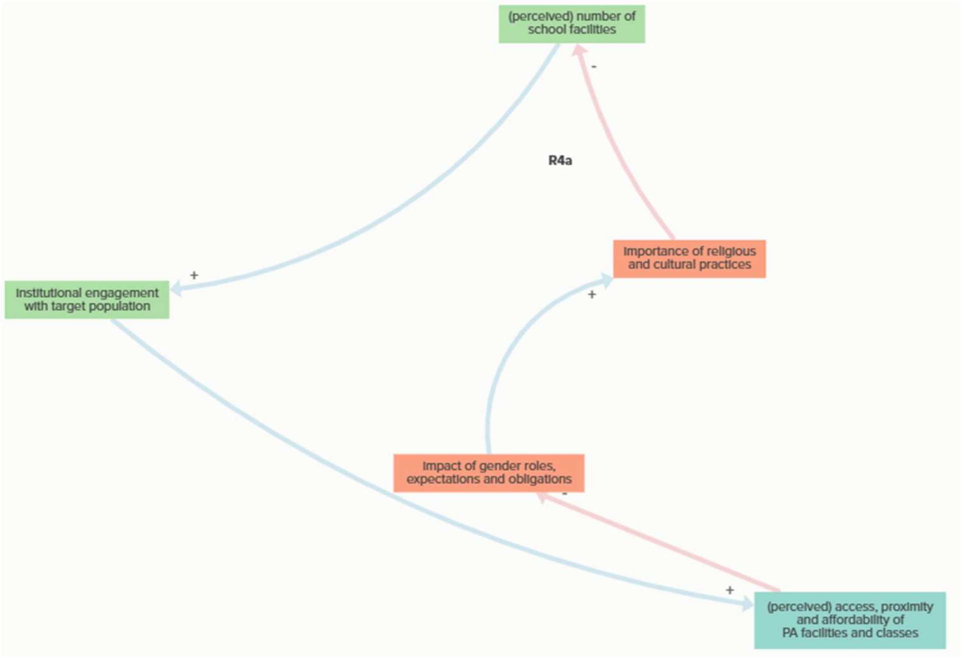

Reinforcing loop R4a: *Lack of appropriate school physical activity facilities reducing access to physical activity opportunities which could reduce the impact of gender roles, expectations and obligations and the religious and cultural practices.* A lack of institutional engagement with the target population, expressed in policies which are not tailored to specific groups, can reduce access to or provision of affordable PA facilities and classes for the whole population. Fewer accessible, local and affordable classes that serve a diverse population could further increase the impact of gender roles, expectations and obligations, as they cannot be used to demonstrate that such facilities and classes are suitable for all women and girls. Greater impact of gender roles, expectations and obligations can increase the personal importance of religious and cultural practices, in turn reducing the number of appropriate school PA facilities if they are deemed not to align with religious teachings or cultural practices. Reduced use of facilities might obfuscate their need and contribute to continued institutional disengagement in schools and related institutions.

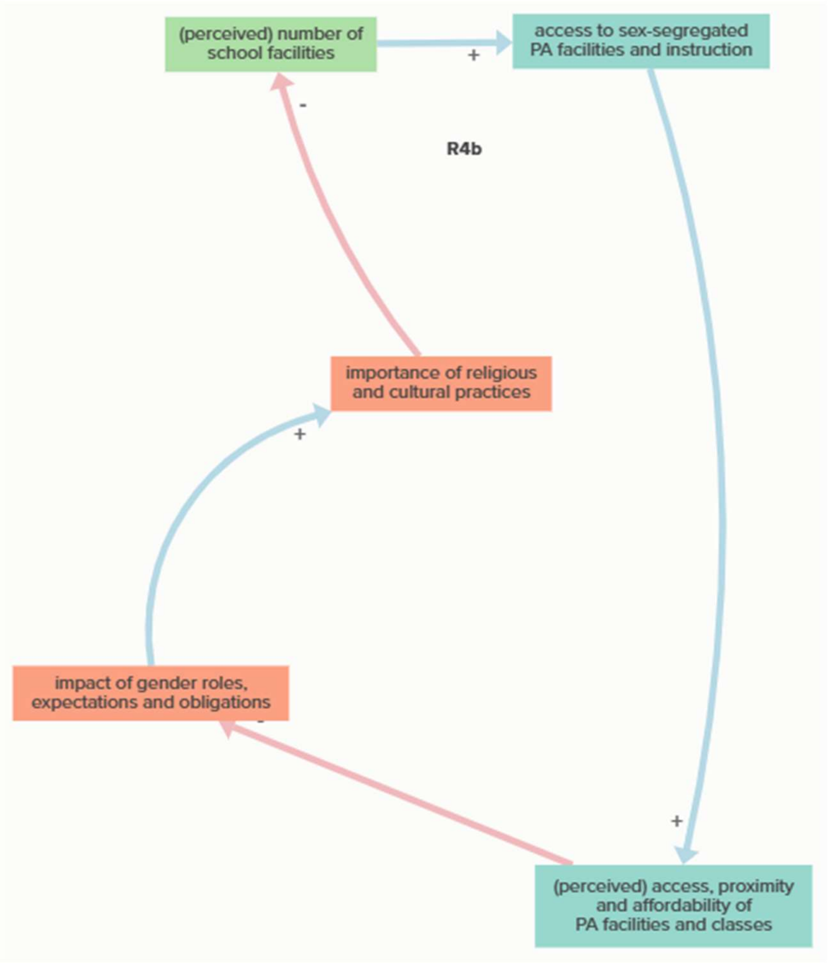

Reinforcing loop R4b: *Lack of appropriate school physical activity facilities reducing access to physical activity opportunities which could reduce the impact of gender roles, expectations and obligations and the religious and cultural practices.* Fewer school facilities for PA limits access to sex-segregated PA facilities and instruction. This can reduce the likelihood of women and girls seeking additional PA facilities and classes in the community, potentially increase the impact of gender roles, expectations and obligations, as such facilities or classes could be perceived as being unsuitable for women and girls. Greater impact of gender roles, expectations and obligations can increase the personal importance of religious and cultural practices which can reduce the number of school PA facilities which are deemed appropriate for girls following certain practices.

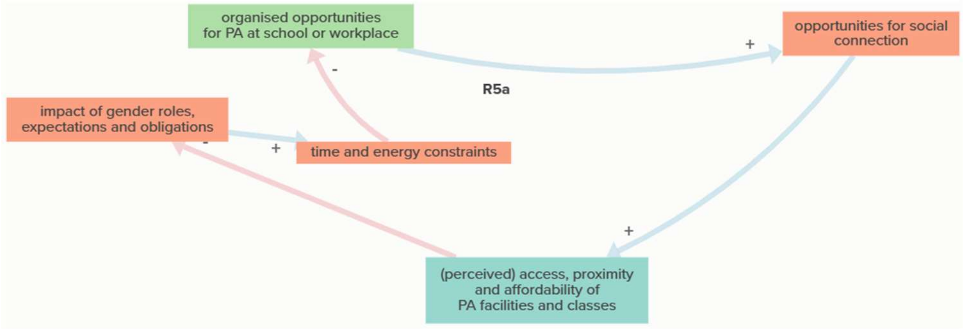

Reinforcing loop R5a: *Greater impact of gender roles leading to reduced perceived access to physical activity facilities, or lower levels of integration, through increased time and energy constraints on women reducing participating in workplace physical activity classes and social connection.* Greater impact of gender roles, expectations and obligations can lead to more time and energy constraints for women who may have more family responsibilities alongside work responsibilities. Such constraints can reduce the opportunity to participate in organised PA in the workplace, leading to missed opportunities for social connection with other PA participants. This can lead to restricted access to PA facilities and classes which may have been shared through that social network, potentially embedding the perception that such facilities are not intended for women and thereby increasing the impact of gender roles, expectations and obligations.

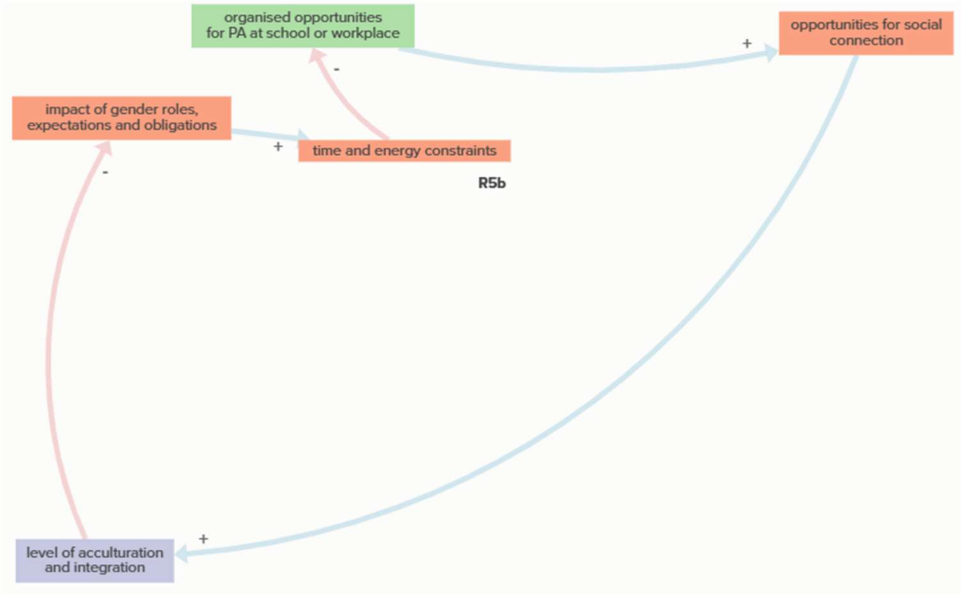

Reinforcing loop R5b: *Greater impact of gender roles leading to reduced perceived access to physical activity facilities, or lower levels of integration, through increased time and energy constraints on women restricting opportunities for workplace physical activity classes and social connection.* Greater impact of gender roles, expectations and obligations can lead to more time and engery constraints for women who may have more family responsibilities alongside work responsibilities. Such constraints can reduce the opportunity to participate in organised PA in the workplace, leading to missed opportunities for social connection with others in the workplace. Lower levels of social connection are likely to impede integration, which can thereby increase the impact of traditional gender roles, expectations and obligations.

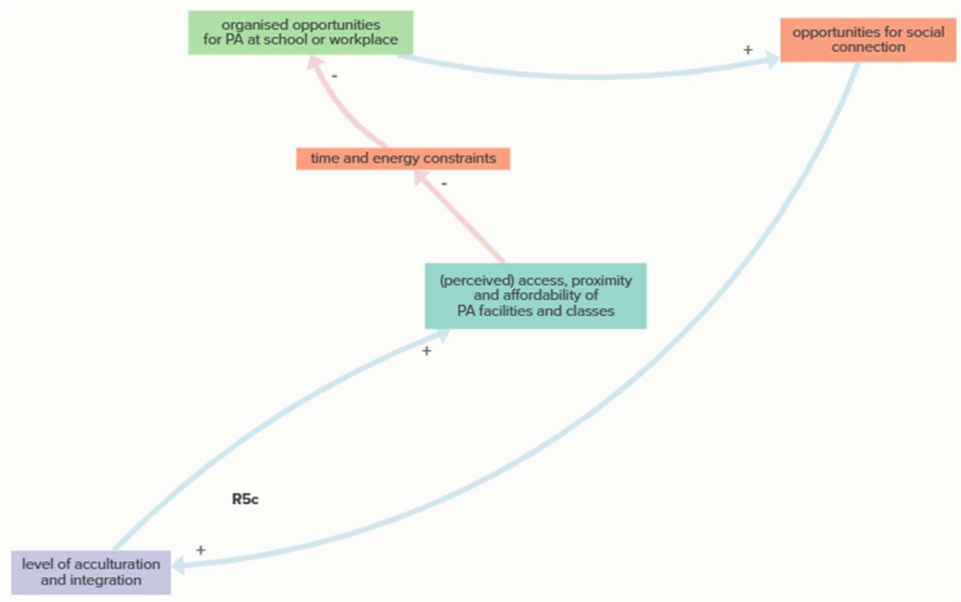

Reinforcing loop R5c: *More organised workplace physical activity classes can lead to reduced time and energy constraints by increasing opportunities for social connection, levels of integration, perceived access of physical activity classes and the time commitments needed to participate in physical activity.* More opportunities to participate in organised PA in the workplace can create new opportunities for social connection with others in the workplace, which can facilitate integration. Higher levels of integration may increase perceived access and actual proximity of workplace and local PA facilities and classes as they may appear welcoming to the individual. This can remove the time and energy commitment needed to go to other physical activity facilities.

Environmental, migration and health and health communication factors sub-system

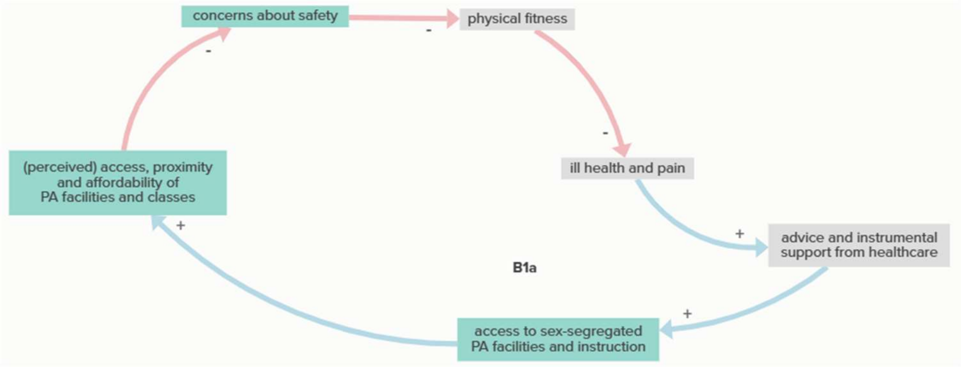

Balancing loop B1a: *Poorer physical and co-morbid mental health can be managed through guided use of appropriate physical activity facilities and instruction.* Higher levels of ill health and pain increase the likelihood of seeking and/or receiving advice and instrumental support for PA from healthcare which can increase access to sex-segregated PA facilities and instruction. In turn, this can increase perceived or actual access of general PA facilities and classes, reducing potential concerns about safety due to an increased sense of familiarity or knowledge of the local area. Fewer concerns about safety can lead to increase physical fitness as individuals feel more able to use the local area for incidental or planned activity, which in turn helps to manage or prevent ill health and pain.

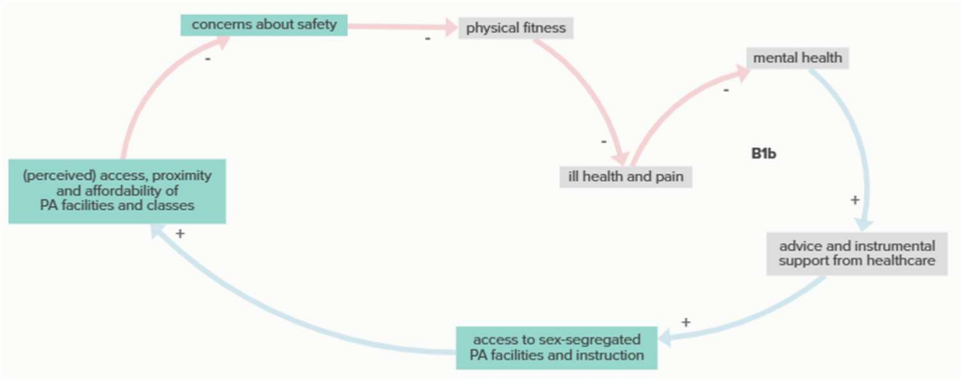

Balancing loop B1b: *Poorer physical and co-morbid mental health can be managed through guided use of appropriate physical activity facilities and instruction.* Higher levels of ill health and pain can contribute to poorer mental health, which can increase the likelihood of seeking and/or receiving advice and instrumental support for PA from healthcare. Advice and support can increase access to sex-segregated PA facilities and instruction. In turn, this can increase perceived or actual access of general PA facilities and classes, reducing potential concerns about safety due to an increased sense of familiarity or knowledge of the local area. Fewer concerns about safety can lead to increase physical fitness as individuals feel more able to use the local area for incidental or planned activity, which in turn helps to manage or prevent ill health and pain.

Environmental, migration and social and material resources factors sub-system

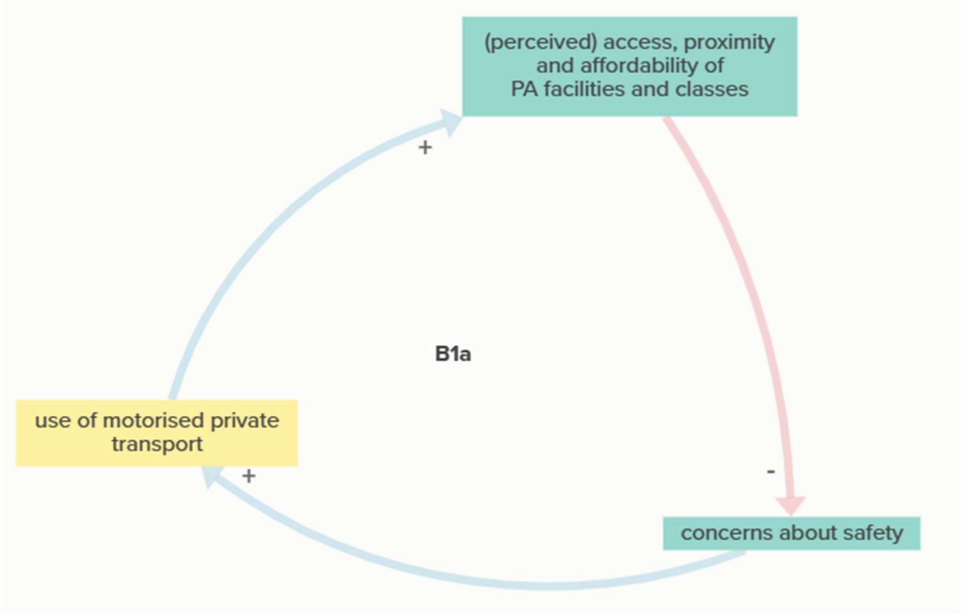

Balancing loop B1a. *The use of motorised transport is sustained due to physical activity facilities being perceived as distant or inaccessible through increased concerns about safety and reduced opportunities for active travel to such facilities.* PA facilities and classes which are (perceived as) inaccessible and far away are more likely to generate concerns about safety in reaching them, due to traffic or unfamiliar settings. Such concerns increase the likelihood of using motorised private transport such as cars, which can increase the perceived accesibility or proximity of PA facilities and classes.

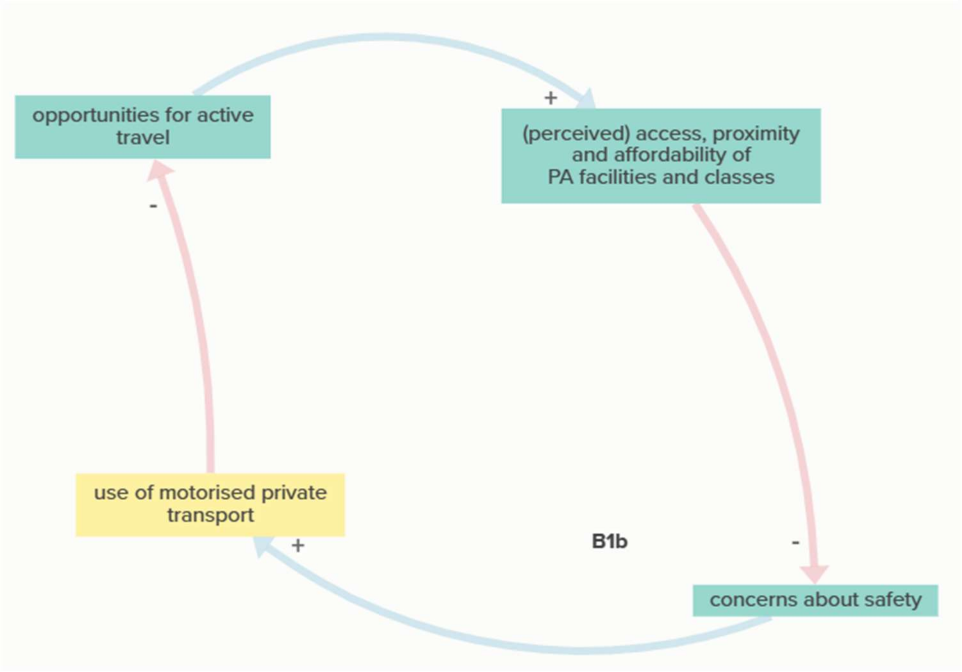

Balancing loop B1b : *The use of motorised transport is sustained due to physical activity facilities being perceived as distant or inaccessible through increased concerns about safety and reduced opportunities for active travel to such facilities.* PA facilities and classes which are (perceived as) inaccessible and far away are more likely to generate concerns about safety in reaching them, due to traffic or unfamiliar settings. Such concerns increase the likelihood of using motorised private transport such as cars, which reduces the opportunities to build active travel into daily living and could otherwise increase the perceived accesibility or proximity of local PA facilities and classes.

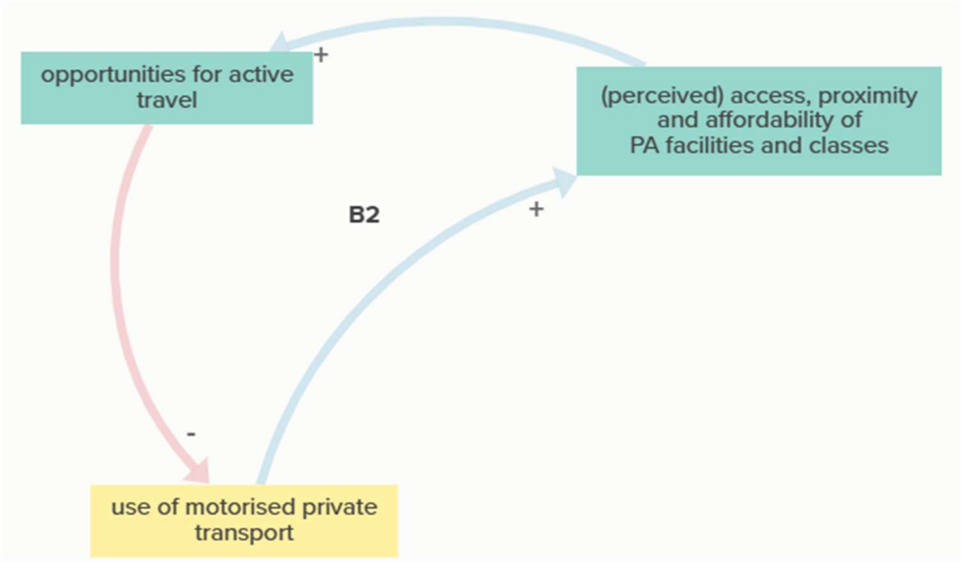

Balancing loop B2: *Low use of active travel to reach physical activity facilities is sustained due to physical activity facilities being perceived as distant or inaccessible and alternative use of motorised transport to reach facilities, where private transport is available.* Lower perceived accessibility or proximity of facilities leads to fewer opportunities to use active travel (including public transportation) to reach facilities, which increases use of motorised private transport, if available, which could increase access to facilities.
